# Interfacial Adsorbate Competition Regulates Intermediate Stabilization and Onset Potential in Acidic CO_2_ Electroreduction

**DOI:** 10.1021/jacs.5c22970

**Published:** 2026-02-26

**Authors:** Adrián Pinilla-Sánchez, Suraj Panja, Bárbara Polesso, Prathama Haldar, Ranit Ram, Ranga Rohit Seemakurthi, Anku Guha, Núria López, F. Pelayo García de Arquer

**Affiliations:** † 172281 ICFO - Institut de Ciències Fotòniques, the Barcelona Institute of Science and Technology, Castelldefels Barcelona 08860, Spain; ‡ Institute of Chemical Research of Catalonia, ICIQ-CERCA, the Barcelona Institute of Science and Technology, Av. Països Catalans 16, Tarragona 43007, Spain; § Department of Physical and Inorganic Chemistry, 202569 Universitat Rovira I Virgili, Campus Sescelades, N4 Block, C. Marcel·lí Domingo 1, Tarragona 43007, Spain

## Abstract

Electrochemical CO_2_ reduction (CO_2_R) in acid may enable high carbon utilization but faces selectivity challenges, particularly from the Hydrogen Evolution Reaction (HER). While the source of protons and cation concentrations play a role in this balance, the role of anions remains underexplored. Here, we combine in situ surface-enhanced Raman Spectroscopy during CO_2_R in acid with theoretical simulations to investigate the role of anionic species over copper gas diffusion electrodes at application-relevant current densities (up to 0.2 A·cm^–2^) and performance. Our observations reveal that sulfate adsorption inhibits CO_2_R at low pH and delays CO_2_R intermediate formation, which is enabled by hydroxyl species coadsorption. Such competition regulates *CO stabilization and the balanced *CO coverage needed to favor the formation of multicarbon products. These results shed light on how anion interactions govern CO_2_R selectivity under acidic conditions and their impact on overpotentials, offering guidance on catalyst–electrolyte interface design.

## Graphical abstract

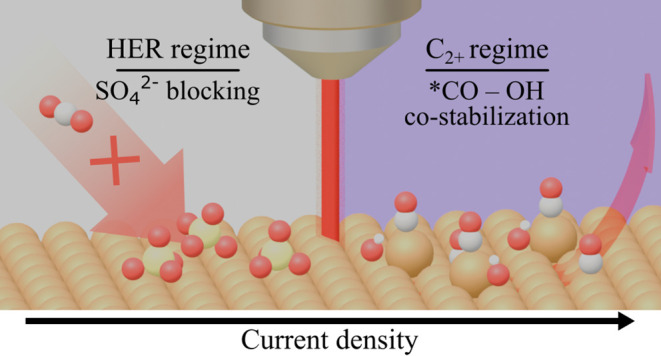

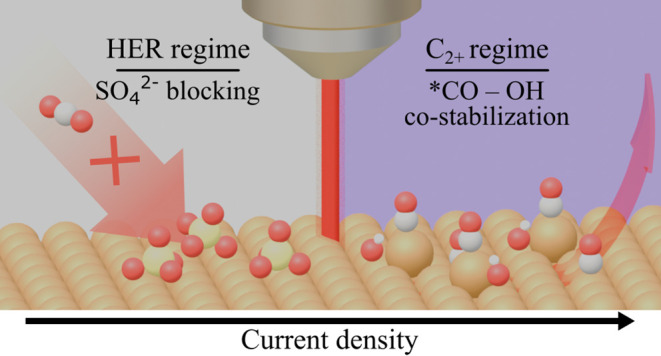

## 1 Introduction

Electrochemical CO_2_ reduction (CO_2_R) offers an attractive approach to transform CO_2_ into high-demand, value-added chemicals. Advances in recent years have improved performance metrics such as selectivity and current density approaching industrial feasibility.^1^ To achieve further progress toward viability, the complete setup needs to ensure sufficient voltage efficiency and stability as well as carbon utilization–limited by parasitic CO_2_ conversion into carbonates by electrolyte and local OH^–^.^2–5^

This prompted research into alternative system designs such as membrane electrode assemblies operated in neutral media, bipolar membranes, tandem configurations, and CO_2_R in acid media.^2,6–19^ The latter offers a way to regenerate carbonates into CO_2_ from protons replenished by the anode and provided through a cation exchange membrane (CEM).

Unfortunately, in proton-rich environments, the CO_2_R is usually dominated by the kinetically favored hydrogen evolution reaction (HER). This is further exacerbated for multicarbon products (C_2+_), which require several electron transfers (*n* = 12 for ethylene vs *n* = 2 for hydrogen). To address this, strategies have focused on modulating the adsorption energies of key adsorbed intermediates such as *CO, *OH, and *H (refs 9–11,14 ) or tuning the local environment—either by restricting H^+^ diffusion^15,16^ or controlling the concentration of cations in the vicinity of the electrode.^13,17–20^

While cations have been extensively studied and recognized for their crucial role in enabling CO_2_R,^17,18,21–38^ the influence of anions remains less understood, particularly in acid media. Emerging evidence suggests that anions are far from being passive species and can actively modulate CO_2_R performance through multiple mechanisms.^39–49^ Among those, bicarbonate and phosphate anions may buffer local pH acting as proton donors, thereby influencing CO_2_R product distribution.^39,49^ This is consistent with theoretical predictions that link buffering capacity to CO_2_ transport limitations.^45^ In other cases, the specific adsorption of anions alters catalyst properties more directly. Large halides have been found to promote CO formation, associated with induced surface restructuring and changes in the electronic structure.^41,42^ Spectroscopic and computational studies further suggest that certain anions, like fluorides or hydroxyl, can modulate *CO binding by modifying the electronic structure of copper.^42–44^

On the other hand, anions like phosphate may poison the surface by occupying active sites, as inferred from changes in *CO binding correlating with phosphate desorption, suggesting its effect by blocking active sites.^40,46^

Altogether, these findings challenge the notion of anions as inert species in CO_2_R. Nonetheless, their mechanistic role—particularly under acidic conditions—remains insufficiently understood. Most of the existing studies have been conducted in neutral or alkaline environments, leaving the behavior and influence of anions in acidic media largely unexplored. This gap is especially pronounced when it comes to direct evidence under operando conditions, highlighting the need for further mechanistic investigations in acidic systems.

In situ surface-enhanced Raman spectroscopy (SERS) has been used to probe the reaction environment, enabling the detection of CO_2_ reduction (CO_2_R) intermediates and adsorbates.^50^ Most of the studies to date have focused on adsorbed *CO and its different adsorption modes, which have been shown to play a crucial role in C_2+_ product formation.^51^ Other key intermediates such as *CO_2_^•–^, *OCH_2_CH_3_, and HOCCO* have also been identified,^52–55^ providing valuable insights into the reaction pathways. In addition, electrolyte anions can also be observed, allowing the estimation of the local pH by tracking the acid–base equilibrium of species (i.e., (bi)­carbonates or phosphates).^56^

Although many early in situ studies focused on simple H-cell configurations producing low current densities (typically a few mA·cm^–2^) and avoiding bubble formation— ^57,58^ more recent work has extended Raman measurements to higher current densities,^59,60^ including in neutral and acidic media.^61^ Since the local microenvironment is known to change significantly between 0 and 100 mA·cm^–2^, it remains crucial to perform in situ measurements under operating conditions to gain meaningful insight into the CO_2_ reduction process.^62^

The primary objective of our work is to achieve a comprehensive understanding and establish control over the local environment of CO_2_R under acidic conditions while concurrently investigating the critical role played by the anion species in this process. To this end, we conducted in situ SERS at high current densities (>200 mA·cm^–2^), using an acid electrolyte composed of 0.5 M K_2_SO_4_ + H_2_SO_4_ adjusted to different pH values. SERS spectra were recorded over a wide potential range (from −0.05 to −3 V_RHE_), enabling us to track the dynamics of anionic species and adsorbed intermediates as a function of both the potential and pH. Clear pH-dependent trends were observed, particularly in sulfate and CO_2_R intermediates, pointing to competitive adsorption that may suppress the availability of active sites. At higher currents, pronounced changes in hydroxide species were also detected, which strongly correlated with the stabilization of the *CO species—a key intermediate in multicarbon product formation. These spectroscopic findings were reinforced by Grand Canonical (GC) DFT simulations and CO_2_R performance experiments, allowing us to mechanistically link the interfacial anion composition to catalytic outcomes. This combined approach provides both fundamental insight into interfacial dynamics and practical guidance for the rational design of acidic CO_2_R systems to multicarbon products.

## 2 Results

Several anion-involved processes can influence the CO_2_R performance at Cu electrode/electrolyte interfaces, such as active site blocking, carbonate-loss-regeneration pathways or CO_2_R intermediate stabilization (Figure 1a). To study the role of anion species in these processes, we performed in situ SERS on a Cu gas diffusion electrode (GDE) coated with perfluorinated sulfonic acid (PFSA) ionomers in an acid electrolyte (0.5 M K_2_SO_4_ + H_2_SO_4_). The different adsorbed species including anions were tracked by in situ Raman spectroscopy at a potential window ranging from preonset potentials (−0.05 V_RHE_/–0.25 mA·cm^–2^) up to peak-performance potentials (−2 to −3 V_RHE_/–100 to 200 mA·cm^–2^) (Figures 1b and 2). Furthermore, to understand how anions dynamically modulate the local environment, we extended in situ SERS measurements across a pH range from 1 to 4.

**Figure 1:**
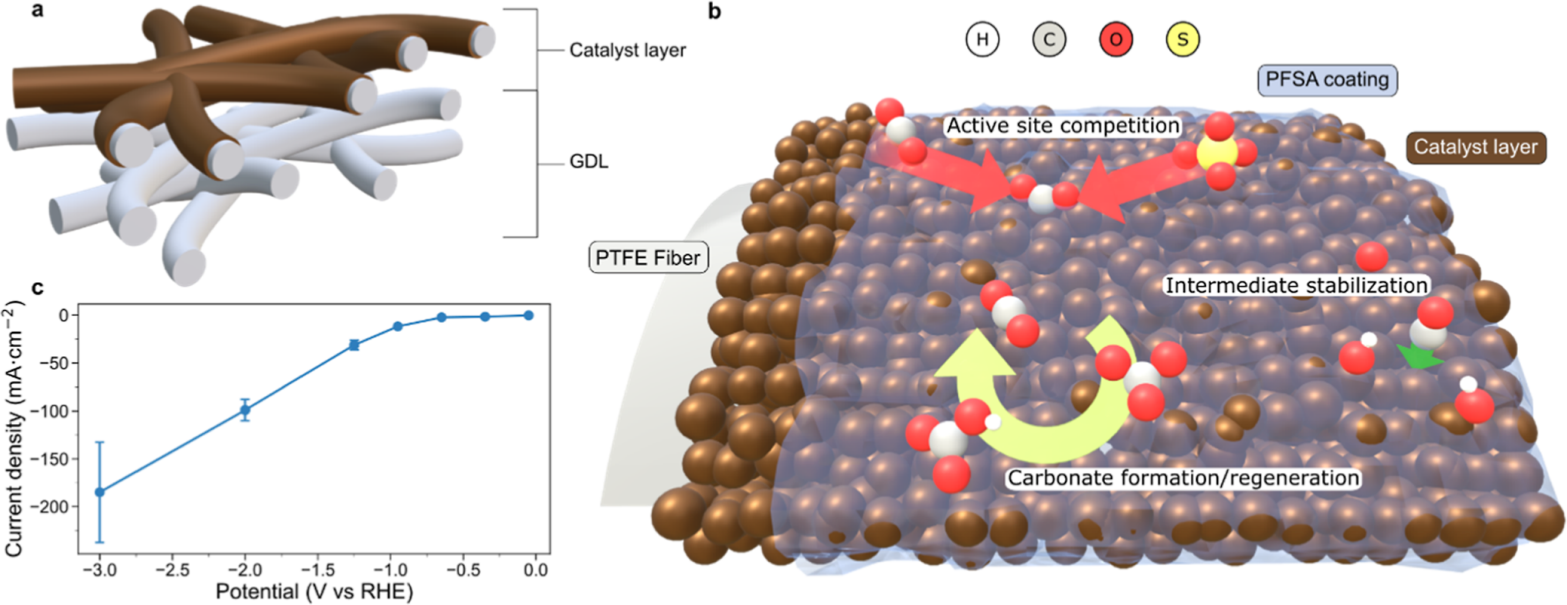
| Strategies to elucidate the role of anions in acid. Schematic of GDE (a) and Cu/PFSA-electrolyte interface (b) showing processes in which anions can be potentially involved in the system: carbonate loss-regeneration pathways, active site blocking, and intermediate stabilization. (c) Representative current density vs. potential curve of in situ Raman spectroscopy experiments up to high current densities.

**Figure 2:**
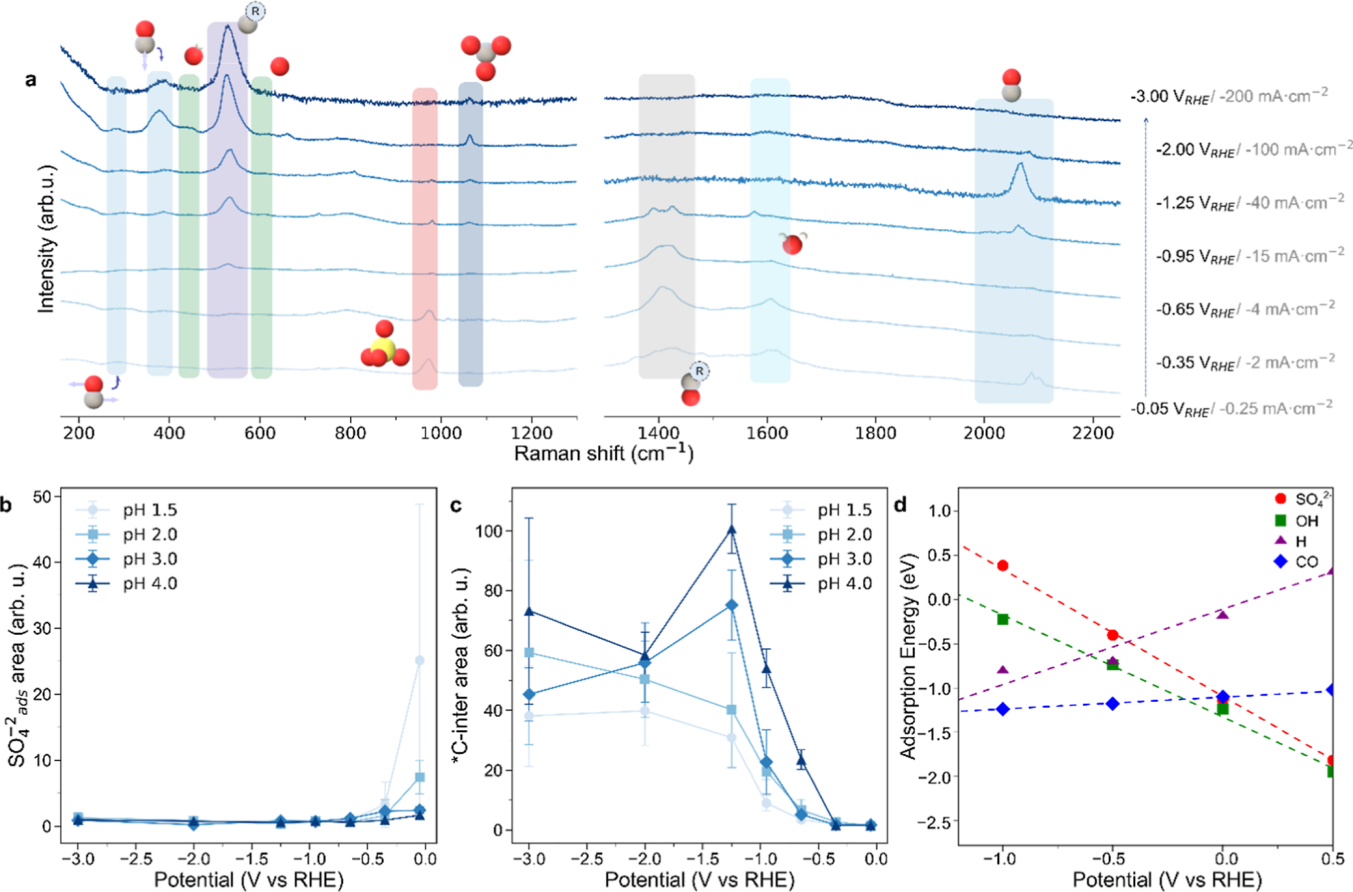
| In situ SERS and DFT insights into competitive anion adsorption. (a) Representative in situ Raman spectra of Cu/PFSA collected by stepping the potential in 0.5 M K_2_SO_4_ + H_2_SO_4_ at pH 2, showing main adsorbed species and intermediates. (b) Integrated area of adsorbed sulfate bands vs potential at various pHs (1.5–4), revealing pH-dependent sulfate adsorption and desorption trends. (c) Integrated area of *C-intermediate bands vs potential at different pHs, showing CO_2_R onset and sulfate-induced inhibition effects. (d) GC-DFT adsorption energies as a function of potential for SO_4_^2–^ (red), OH^–^ (green), H^+^ (purple), and CO (blue) on Cu(100), highlighting competitive anion adsorption and desorption trends.

### 2.1 Low Current Regime: Anion Blocking of Active Sites

At low overpotentials, in situ Raman spectra reveal distinct features at 980 and 970 cm^–1^ (Figure S9). The 10 cm^–1^ redshift of the 970 cm^–1^ band is consistent with surface adsorption, and we therefore attribute these bands to free and adsorbed sulfate, respectively (Figures 2a,b and S9, Table S1, Note S1).^63–65^ A feature appears at 1405 cm^–1^ which is assigned to a mixture of ionomer and *O–C–(X) species (Figure S8),^1,55^ along with minor contributions from bicarbonate bands at 1020 and 1356 cm^–1^, which are also observed at −0.05 V_RHE_ but disappear at −0.35 V_RHE_.^66^ Additionally, bands appearing in the 1800–2100 cm^–1^ region, attributed to adsorbed CO species, display a dominating stochastic and strongly time-dependent behavior (Figures 2a and S18), most likely arising from surface reconstruction processes taking place within this potential range.^67,68^

Desorption of sulfate at −0.65 V_RHE_ coincides with the appearance of a band around 530 cm^–1^ (Figure 2a, c), which we attributed to carbon-containing CO_2_R intermediates (*C-intermediate) based on isotope labeling experiments (Figure S7). The origin of this band remains a matter of debate, with some studies associating this band to hydroxyl/oxide species^44,69–73^ while others assigning it to carbon-related intermediates,^74–78^ or a mixed contribution of different CO_2_R intermediates,^74^ or hydroxyl species and intermediates.^11,77,79^

The persistence of sulfate adsorption at low overpotentials coupled with the absence of CO_2_R intermediates until desorption suggests that sulfate anions are blocking active sites at low overpotentials, preventing CO_2_ adsorption and intermediate formation, until sufficient negative potentials weaken their adsorption and enable CO_2_R.

This behavior mirrors findings from phosphate-containing electrolytes, where anion blocking of Cu surfaces was shown to limit *CO adsorption.^40,46^ We hypothesized that sulfate anions might be similarly poisoning the surface at low potentials in a way similar to that of phosphate, impeding the adsorption of CO_2_ and the formation of intermediates. Supporting this, pH-dependent experiments show that at lower pH values (pH 1.5–2), stronger sulfate adsorption delays the onset of the *C-intermediate until more negative potentials compared to higher pH values (pH 3–4 and K_2_SO_4_ control), where weaker adsorption allows earlier intermediate formation (Figures 2b,c and S5).^80^ This pH-dependent behavior is consistent with competitive hydroxyl-sulfate adsorption,^81–83^ where hydroxyl displacement of sulfate species restores active site accessibility and enables earlier *C-intermediate formation.

### 2.2 Competing Anion Adsorption: Insights from GC–DFT Simulations

To further elucidate competitive adsorption dynamics, we employed GC–DFT simulations using the PBE-D2 functional to investigate the interplay among CO, protons, hydroxyl, and sulfate adsorption on the most stable copper facets^84^ Cu(100) and Cu(111), as a function of applied potential (Figure 2d).

The adsorption trends reveal that near 0 V_RHE_, sulfates and hydroxides adsorb strongly compared to CO and protons, blocking the sites for CO_2_R. Under these strong acidic conditions (pH 0–2), hydroxide concentration is negligible compared to sulfate anions, which can be considered the primary site blocking species at low overpotentials. However, at higher pH values (pH 3–4), the increasing concentration of hydroxyls would be expected to displace sulfate adsorption,^81–83^ a trend consistent with the pH-dependent behavior observed (Figures 2b and S9).

As the potential becomes increasingly negative, the binding strength of sulfate and hydroxide species weakens, this weakening being more pronounced for sulfate due to its higher negative charge, which enhances electrostatic repulsion at negative biases. This relative change in adsorption energies leads to a shift in surface coverage, where at more negative potentials (<−0.4 V_RHE_), the adsorption of CO becomes more favorable, resulting in the desorption of sulfate anions. These results are in line with experimental trends in sulfate, hydroxyls, and *CO bands, supporting the hypothesis of active site blocking by adsorbed sulfate anions.

Overall, these DFT and experimental results confirm that in the low-current regime (<15 mA·cm^2^), sulfate adsorption limits CO_2_R by blocking active sites and inhibiting CO_2_–CO adsorption. The transition to CO_2_R-active conditions requires sulfate desorption, a process that depends on both the potential and bulk pH value.

### 2.3 Surface Reconstruction and CO Adsorption at Medium Currents

Upon sulfate desorption, CO_2_R can start, as indicated by the emergence of the *C-intermediate band and *CO-related Raman features at 290 and 385 cm^–1^, which can be assigned to frustrated Cu–CO rotation and Cu–C­(O) stretching. Adsorbed CO in atop and bridge configurations contributes to these peaks but with different relative intensities. Atop *CO gives a relatively stronger stretching contribution and weaker rotational features, whereas bridge *CO leads to a more pronounced rotational component. This establishes the str/rot CO ratio as a qualitative descriptor of the relative population of atop versus bridge *CO species, which has been shown to be directly correlated with *CO coverage and C_2+_ products selectivity.^85^

As the potential increases to −0.35 V_RHE_, a slight increase in the str/rot CO ratio is observed, probably associated with the desorption of sulfate anions that favors the adsorption of CO (Figure 3a,b). But as potential further decreases, the str/rot *CO ratio slightly decreases until −0.95 V_RHE_/40 mA·cm^–2^, suggesting an increased stabilization of *CO in bridge configurations. A similar trend can be observed on the 450 cm^–1^, assigned to *OH in bridge configuration (Table S2),^79,86^ suggesting possible competition with emerging carbon intermediates and carbonate species (Figure S12) or bridge-bound *CO.

**Figure 3:**
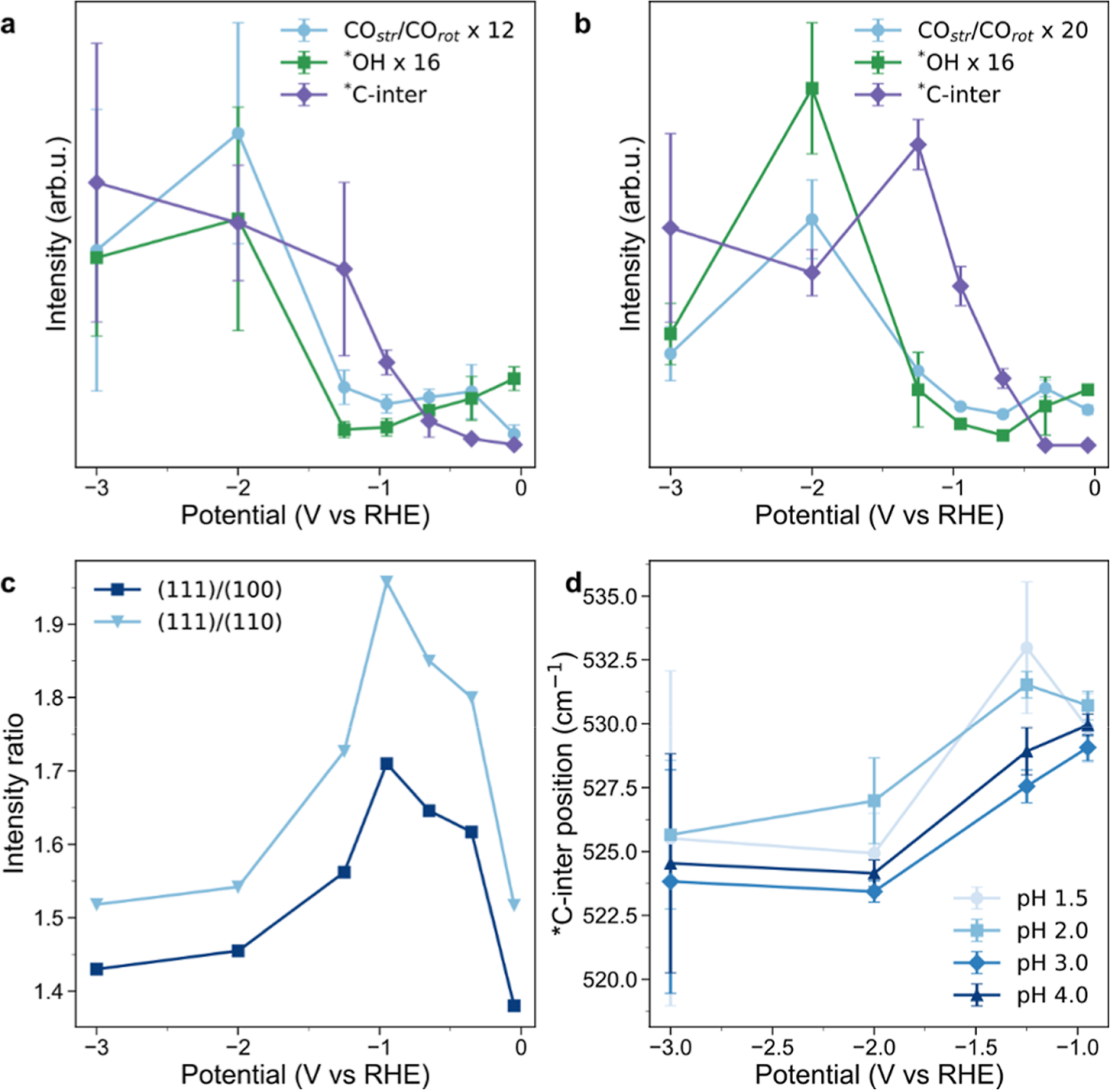
| Anion effects on CO_2_R intermediates and Cu surface evolution at high current density. (a,b) Comparative analysis of CO_2_R-relevant species at pH 2 (a) and 4 (b), with scaled intensity of *C-intermediate (purple), str/rot CO band ratio (blue), OH_bridge_ (green) vs potential. (c) XRD-derived intensity ratios of Cu(111) relative to Cu(100) (dark blue) and Cu(110) (light blue), showing potential-induced surface restructuring. (d) *C-intermediate band position as a function of potential.

### 2.4 High Current Regime: Synergistic OH–CO Stabilization Drives C_2+_ Selectivity

Below −1 V_RHE_, *CO coverage increases, as seen from the str/rot CO ratio increase, peaking around −2 V_RHE_ (Figures 3a,b and S16). This aligns with fast ex situ XRD measurements, which reveal a crystalline structure change at potentials below −1 V_RHE_, favoring Cu(100) and Cu(110) facets over Cu(111) (Figures 3c and S19). These facets bind *CO more strongly, potentially altering the *CO adsorption configuration and coverage.^40,87^

Notably, values of the str/rot CO ratio at −2 V_RHE_ are higher than those previously reported in literature,^85^ indicating a particularly high proportion of atop-bound *CO under our experimental conditions. Interestingly, the relationship between *CO coverage and potential is influenced by pH. At low pH values (pH 1.5–2), a higher *CO coverage is achieved compared to higher pH values (pH 3–4). This enhanced *CO formation at lower pH might be explained by a higher proton availability, which promotes the first proton-coupled electron transfer (PCET) steps and promotes *CO formation.^88,89^

Concurrently, marked increases are observed in the 450 and 605 cm^–1^ bands associated with *OH_bridge_ and adsorbed oxide species.^69^ The increase of str Cu–O and carbonate bands (Figure S14) at increased negative potentials reflects an increase in local alkalinization at high current densities.^90^

A strong positive correlation is evident between *OH_bridge_ and str/rot CO ratio (Figures 3a,b, S16), suggesting a potential synergistic interaction between *OH_bridge_ and *CO. At lower pHs, the increased proton availability enhances *CO formation through PCET steps while limiting OH^–^ availability, while at higher pH, the surface becomes more hydroxylated due to increased water dissociation, which stabilizes *OH_bridge_ but reduces *CO coverage. These trends suggest local pH effects might play a critical role in modulating the balance between *OH_bridge_ and *CO,^86^ particularly under acidic conditions.

The observed correlation between *OH_bridge_ and *CO suggests that *OH_bridge_ may stabilize *CO adsorption, or vice versa. The origin of this stabilization, however, is not clear and could arise from different factors. It may result from direct interaction between *OH_bridge_ and *CO,^10,91^ or from indirect effects, such as changes in the electronic structure or surface reconstruction induced by *OH or *CO adsorption.^44,87^ To probe this further, DFT simulations were run at high *CO coverages (0.77 ML) on Cu(100), revealing that *OH adsorption becomes less favorable by 0.97 eV as compared to a clean slab. However, at such high *CO coverages, adatom formation is known to be feasible.^92^ Our simulations show that these adatom configurations (Figure S29) indeed facilitate the coadsorption of *CO and *OH with a exothermic adsorption energy of −0.74 eV at 0 V_RHE_. However, this is in competition with *CO–*CO geminal structures, which have an adsorption energy −0.34 eV and are feasible especially at high *CO local concentrations. Thus, the adsorption of *OH and *CO on adatom structures is in dynamic competition, influenced by interfacial conditions (pH and potential). Furthermore, the linear correlation between *OH and *CO peaks become stronger at higher pH (Figure S16), supporting the hypothesis that alkaline conditions promote coadsorption.

At high current densities, changes are also observed in the *C-intermediate band, marked by a redshift at −2 V_RHE_ (Figure 3d), indicating a change in the coordination environment of these intermediates. This redshift coincides with the potential at which *CO coverage—associated with C_2+_ product selectivity—^85^ reaches its maximum (Figures 3a,b and S16). This supports the idea that the *C-intermediate band is linked to the C–C coupling step in the CO_2_R. However, the rise of this band occurs prior to the major increase in *CO coverage, implying that early intermediates in the CO_2_R pathway—or other species such as hydroxyl/oxides—may also contribute to this peak.^74^ To identify the specific species contributing to this band, we have taken the configurations of several C_1_ and C_2_ candidate intermediates on Cu(100) with the predicted vibrational frequencies in the range of 510–550 cm^–1^ from our previous work, Pablo-García et al.^93^ We then performed theoretical Raman spectra analysis of these intermediates to find that only *CHCO and *CH_2_OH have high intensities in this regime (Figures S30 and S31, Tables S1 and S6). Based on the combined insights from isotope-labeling experiments and DFT simulations, we conclude that *CHCO and *CH_2_OH are the most likely contributors.

### 2.5 Electrochemical Performance

To identify possible correlations between in situ SERS trends and CO_2_R performance, CO_2_R studies were conducted in the same electrolytes as in the spectroscopic studies. Experiments were performed in galvanostatic mode, and currents were selected based on SERS results at different pHs. The selected range was from 15 mA·cm^–2^ (equivalent to −1.25 V_RHE_, Figure S20), just above the onset potential of *C-intermediate bands and sulfate desorption, up to 500 mA·cm^–2^.

Faradaic efficiencies toward H_2_, C_1_, and C_2+_ products show a clear pH-dependence of onset potential for CO_2_R in the low current range (Figure 4a–d). Under more acidic conditions (pH 1.5–2), H_2_ is the main product at lower current densities (15 mA·cm^–2^). In contrast, at higher pH values (pH 3–4), C_1_ and some C_2+_ products start to be observed at the same current density, reaching significant FE > 40%. This pH-dependent CO_2_R onset correlates with the pH-sensitive adsorption of sulfates (Figure 2b,c), which might be poisoning the catalyst and inhibiting CO_2_R.^40,46^

**Figure 4:**
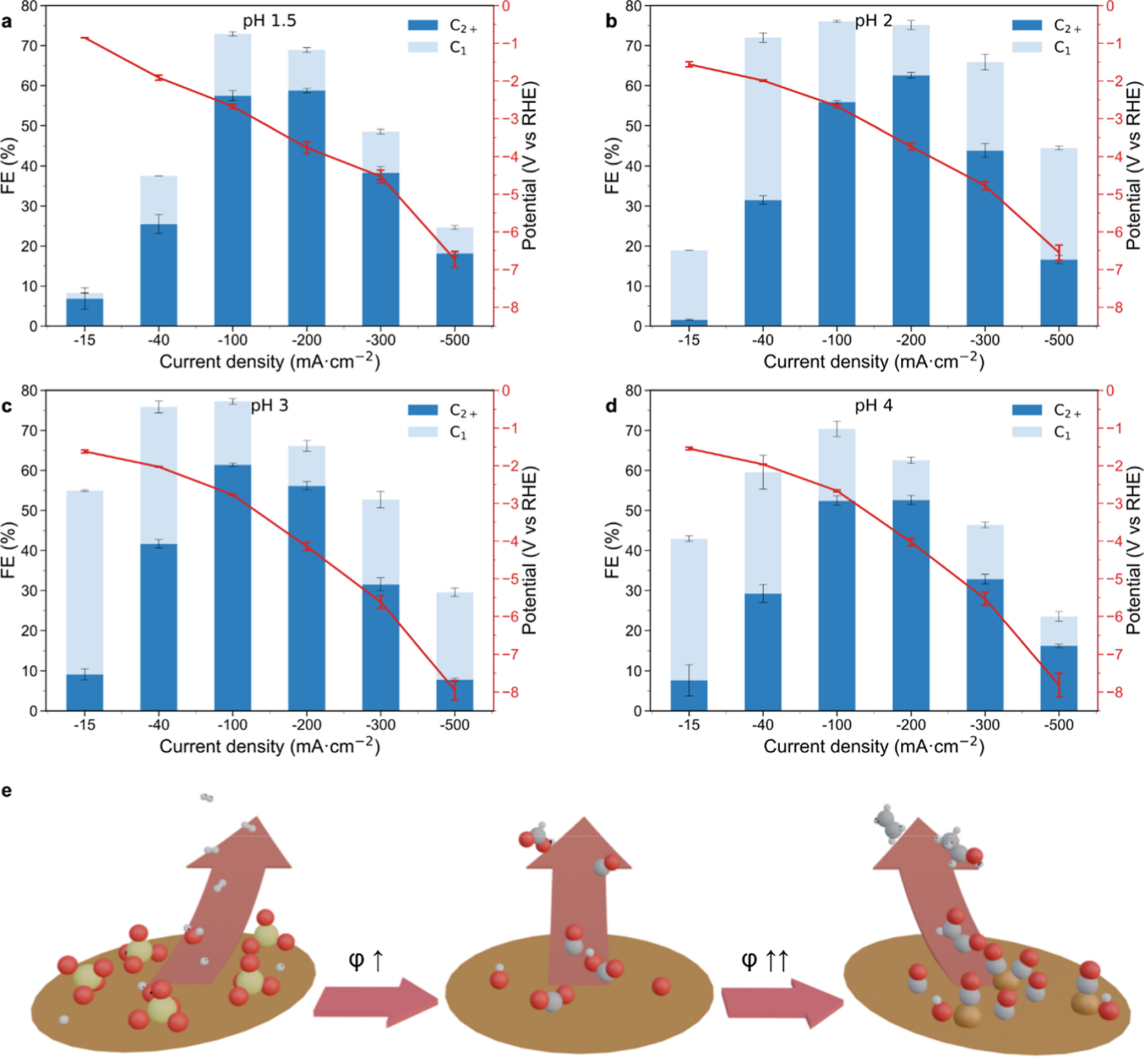
| Acidic CO_2_R performance. (a–d) Faradaic efficiency toward H_2_, C_1_, and C_2+_ products at different current densities and pHs. (e) Schematic of ongoing processes in the potential range of in situ experiments, showing poisoning of the surface by sulfate anions at low current densities and stabilization of the *CO intermediate at increasing potential (φ), enhanced by hydroxyl/oxide species.

Moreover, when the pH is lowered to 1, no stable CO_2_R products can be observed even at higher current densities (Figure S23). This aligns with in situ SERS data at pH 1 (Figure S1), which shows no *C-intermediate or *CO intermediate bands in the potential region evaluated. At potentials less negative than −1 V_RHE,_ Raman spectra could not be acquired due to intense bubble evolution from the HER. Thus, stabilization of *C-intermediates and *CO species at more negative potentials cannot be ruled out.

With increasing current densities, selectivity gradually shifts from C_1_ to C_2+_ products, regardless of the pH, becoming dominated by C_2+_ selectivity at 100 mA·cm^–2^. This transition coincides with the str/rot *CO band ratio maximum and the shift in the *C-intermediate band at −2 V_RHE_/100 mA·cm^–2^, as observed during the in situ experiments. At current densities above 100 mA·cm^–2^, the str/rot *CO ratio strongly decreases in the in situ experiments, which may be related to the faster CO_2_R kinetics observed in this regime. Moreover, the higher *CO coverage detected during the in situ experiments at more acidic pH values (pH 1.5–2) (Figures 3a,b and S16) correlates with the enhanced performance toward C_2+_ products at the high current density range (300–500 mA·cm^–2^), compared to the higher pH electrolytes (pH 3–4) (Figure 4a–d).

Figure 4e summarizes the events that occur in a concerted manner on the electrode surface at a decreasing potential. At low potentials, surface is blocked by adsorbed sulfate species; thus, only HER happens in this potential range. Around −0.65 V_RHE_, sulfate species begin to desorb, allowing for the adsorption of CO_2_ and formation of CO_2_R intermediates (Figure 3a,b). This process determines the onset potential of CO_2_R and is influenced by the bulk electrolyte pHs: at more acidic pH, the sulfate coverage is higher and poisons the surface up to higher potentials, delaying the onset of CO_2_R (Figure 4a–d).

As the potential further decreases and *CO accumulates on the surface, formation of adatoms in the surface starts to happen. These Cu adatoms configurations facilitate the coadsorption of *CO and *OH. This coadsorption likely plays a role in stabilizing *CO on atop configuration, leading to higher *CO coverages and promoting CO_2_R selectivity toward C_2+_ products around −2 V_RHE_/100 mA·cm^–2^. The accumulation of *CO is enhanced at more acidic pHs, which gets translated also into better performance toward C_2+_ products at higher current densities (Figure 4a–d).

## 3 Conclusions

This study reveals that anions play an active and dynamic role in steering the CO_2_R on Cu/PFSA electrodes under acidic conditions and industrially relevant conditions. By integrating in situ SERS with DFT simulations, we show that at low overpotentials, strongly adsorbed sulfate anions block active sites, delaying the formation of key CO_2_R intermediates and thereby regulating the onset of catalysis in acidic media. As the potential becomes more negative, *CO and bridge-bound *OH species coadsorb in the surface, suggesting a cooperative stabilization mechanism that enables higher and more balanced *CO coverage. This shift in interfacial chemistry directly correlates with enhanced C–C coupling and increased C_2+_ product formation.

These findings highlight the importance of precisely tuning interfacial species to shift selectivity toward multicarbon products, redefining the role of anions in acidic CO_2_R and positioning them as strategic actors for interface engineering. Furthermore, our study emphasizes the necessity of conducting in situ experiments at performance-relevant current densities, particularly in strong acid environments, where CO_2_R onset occurs at higher current densities.

## 4 Experimental Section

### 4.1 Gas Diffusion Electrode Preparation

Cu electrodes, with a thickness of 300 nm, were prepared by sputtering pure Cu onto a PTFE gas diffusion layer with a 450 nm pore size. Cu/Perfluorosulfonic acid ionomer (PFSA) electrodes were prepared by spray coating 2 μL·cm^–2^ of Aquivion^TM^ (25 wt %, Sigma-Aldrich) dispersed in methanol (99.9%, Scharlau).

### 4.2 In Situ Raman Spectroscopy Measurements

Raman spectra were obtained in a Renishaw inVia spectrometer, equipped with a 785 nm wavelength laser (200 mW), an 1800 lines/nm grating, and immersion objective (L63×, Leica). In order to avoid objective corrosion, a 20 μm PVC film was used to protect it. Laser was attenuated to 0.5% power to avoid sample damage. Spectra were collected in three different wavenumber windows 150–1300 cm^–1^, 1300–2300 cm^–1^, and 2400–3100 cm^–1^. Spectra were acquired with 25 acquisitions with 2s exposition each. A minimum of three spectra per potential and wavenumber window were acquired in order to ensure reproducibility.

In situ Raman spectroscopy experiments were carried out in a homemade PTFE electrochemical cell consisting of two compartments, a gas chamber and an electrolyte chamber. Area of working electrode was restricted to 0.5 cm^2^. Pt wire and Ag/AgCl (1 M KCl) were used as the counter and reference electrode, respectively. Electrolyte used for the experiments consisted on 0.5 M K_2_SO_4_ + H_2_SO_4_ adjusted to different pHs ranging from pH 1 to 4. CO_2_ gas flow of 40 standard cubic centimeters per minute (sccm) was supplied in the gas chamber.

The potentials were applied using a SP-50e Bio-Logic SAS potentiostat model controlled by EC-Lab software. Before introducing the electrolyte, the sample was held at −100 μA·cm^–2^ to avoid sample oxidation before applying the potentials. Constant potentials from −0.05 to −3 V_RHE_ were applied to the sample for a minimum of 200 s before acquiring the spectra.

Raman bands were analyzed using Python v3.12 and NumPy, pandas, matplotlib, SciPy, and pybaselines modules, using the same procedure for all the over 120 spectra. All spectra were first normalized to the background signal to account for differences in signal intensity arising from variations in laser focus. This normalization approach was validated by the reduced peak amplitude variations observed across consecutive measurement points, confirming that intensity fluctuations in our data set originate primarily from focal depth differences. Baseline subtraction was carried out using the “Iterative morphological and mollifier-based baseline correction method”^94^ to remove background contributions. Resulting spectral bands were subsequently fitted by using Gaussian functions. All peaks were fitted with single Gaussian functions, except for the *C-intermediate peak, which was fitted with three Gaussian components^11^ to properly account for peak shoulders (Figure S13) and the sulfate peak, which was decomposed into two Gaussian components to resolve adsorbed and free sulfate species^63^ (Figure S15). Peak amplitude, full width at half-maximum (fwhm), and peak position were extracted from the fitting procedure, while the integrated Area values are derived from the area under each fitted Gaussian curve. Error bars presented in graphs arise from standard deviation between fitting parameters among different measurements (3–6 measurements per condition and spectral window).

### 4.3 Electrochemical CO_2_R Performance Measurements

The CO_2_R performance measurements were carried out in a three-compartment flow cell using an electrochemical workstation (Autolab204.s) connected to a current booster in galvanostatic mode. Anolyte and catholyte chambers were separated by a Nafion^TM^ 117 membrane. Pt mesh and Ag/AgCl (3 M KCl) were used as counter and reference electrodes, respectively. For all experiments, anolyte was 0.5 M H_2_SO_4_, while catholyte was 0.5 M K_2_SO_4_ + H_2_SO_4_ adjusted to different pHs ranging from pH 1 to 4. A fixed volume of 20 and 40 mL for catholyte and anolyte was recirculated through anode and cathode compartments, respectively. Electrolyte flow rate was fixed to 30 mL·min^–1^ for both anolyte and catholyte and CO_2_ flow used was 40 sccm.

Gas products from CO_2_R were analyzed using a gas chromatograph (PerkinElmer Clarus 590) coupled with a thermal conductivity detector (TCD) and a flame ionization detector (FID). Argon was used as the carrier gas. A minimum of three injections were performed at every current density. Error bars arise from the standard deviation between injections.

Liquid products were analyzed using a High-Pressure Liquid Chromatography (HPLC) (Agilent 1220 Infinity II) instrument coupled with a Diode Array Detector (DAD, 194 and 210 nm) and Refractive Index Detector (RID). Liquid product was collected and injected once per current and after every third injection in the GC.

### 4.4 Computational Details

DFT simulations were carried out using Vienna Ab initio Simulation Package^95,96^ (VASP 5.4.4). DFT-D2 method using our reparametrized C_6_ coefficients for metals was applied to account for the long-range van der Waals interactions.^97^ Core electrons were represented using projected Augmented Wave (PAW)^98^ with the Perdew Burke Ernzerhof (PBE)^99^ functional. The valence electrons were expanded in plane waves with a kinetic energy cutoff of 450 eV. The converged bulk lattice constant of Cu was 3.63 Å. Cu(100) and Cu(111) were constructed as a (4 × 4) supercell with 5 layers with a vacuum of 12 Å in the *z* direction. Γ-Centered *k*-point mesh of 3 × 3 × 1 was used. To determine the partial electron occupancies, Gaussian smearing scheme was used with an energy smearing of 0.03 eV. The electronic convergence criteria were set to 10^–5^ eV, and the structures were converged until the forces were below 0.03 eV/Å. Numerical frequencies were computed by finite displacements with a step size of ±0.015 Å All simulations were nonspin polarized, and dipole corrections were applied in the *z* direction. The gas-phase molecules (H_2_SO_4_, H_2_O) were simulated in a cubic box of 10 Å, and CO gas phase energy was corrected with respect to CH_4_.^100^ To account for electrochemical steps, Computational Hydrogen Electrode^101^ approximation was used, while anion energy was estimated using p*K*_a_ values. The detailed derivation of the binding energies can be found in the Supporting Information. Implicit solvation corrections were calculated using VASP-MGCM (VASP-Multigrid Continuum Model).^102^ All of the structures were simulated such that the anions accept electrons from the surface. Three most stable configuration sulfates and their binding energies are shown in Figures S24 and S25 and Tables S3 and S4. For the most stable structures for these intermediates, we applied the Grand canonical formalism using VASP-sol++^103^ to estimate the binding energies as a function of potential, as shown in the Figure S28. The Raman active modes were calculated using phonopy spectroscopy package.^104^

## Supplementary Material



## Data Availability

DFT simulations have been uploaded to the iochem-BD database (10.19061/iochem-bd-1-380).

## Notes

Additional in situ Raman measurements with spectral feature fitting, supplementary electrochemical and materials characterization analyses, and expanded DFT simulations including detailed procedures for adsorption energy calculations (PDF)
