# Supplementary material for: Interfacial Adsorbate Competition Regulates Intermediate Stabilization and Onset Potential in Acidic CO2 Electroreduction
Source: J Am Chem Soc. 2026 Feb 26;148(9):10026–36. doi: 10.1021/jacs.5c22970 (PMC12983304; doi:10.1021/jacs.5c22970)
Supplement: Supplementary file 1 [file ja5c22970_si_001.pdf]

Interfacial Adsorbate Competition Regulates Intermediate Stabilization and Onset Potential in Acidic CO_2 Electroreduction

Adrián Pinilla-Sánchez[1], Suraj Panja[2,3], Bárbara Polesso[1], Prathama Haldar[1], Ranit Ram[1], Ranga Rohit Seemakurthi,[2] Anku Guha[1], Núria López,[2,*] F. Pelayo García de Arquer[1,*]

[1]ICFO - Institut de Ciències Fotòniques, The Barcelona Institute of Science and Technology, Castelldefels (Barcelona), 08860, Spain
[2]Institute of Chemical Research of Catalonia, ICIQ-CERCA, The Barcelona Institute of Science and Technology, Av. Països Catalans 16, 43007 Tarragona, Spain
[3]Department of Physical and Inorganic Chemistry, Universitat Rovira i Virgili, Campus Sescelades, N4 Block, C. Marcel·lí Domingo 1, Tarragona, 43007, Spain.
*corresponding authors: nlopez@iciq.es, pelayo.garciadearquer@icfo.eu

In situ SERS at different pHs

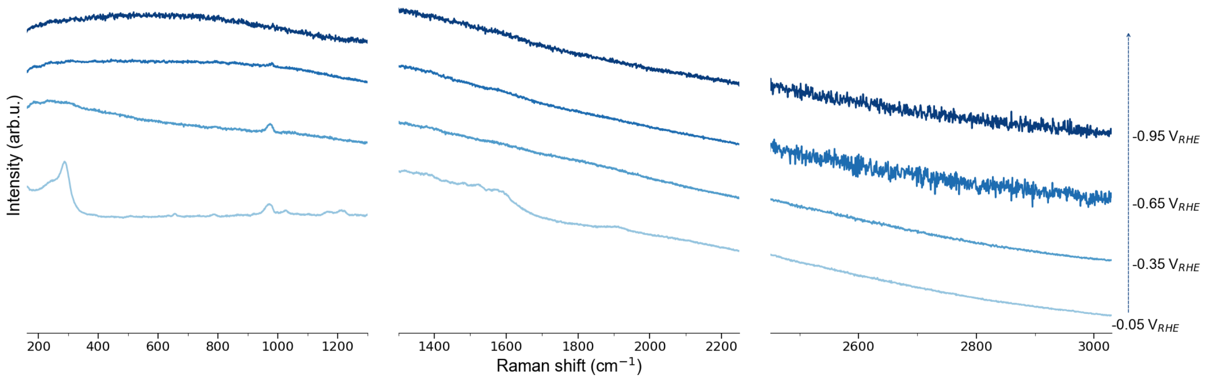

Figure S1: In situ Raman spectra of Cu/PFSA at different potentials in 0.5M K_2SO_4 + H_2SO_4 at pH 1.

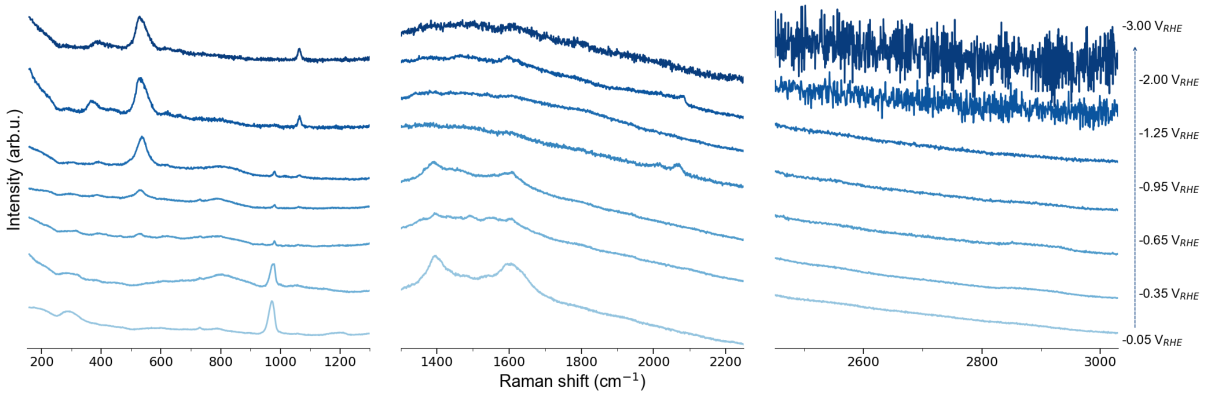

Figure S2: In situ Raman spectra of Cu/PFSA at different potentials in 0.5M K_2SO_4 + H_2SO_4 at pH 1.5.

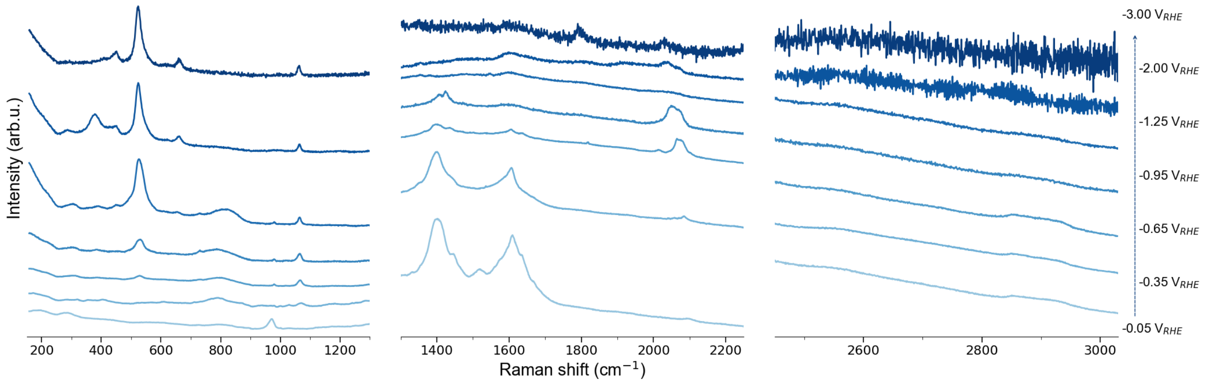

Figure S3: In situ Raman spectra of Cu/PFSA at different potentials in 0.5M K_2SO_4 + H_2SO_4 at pH 3.

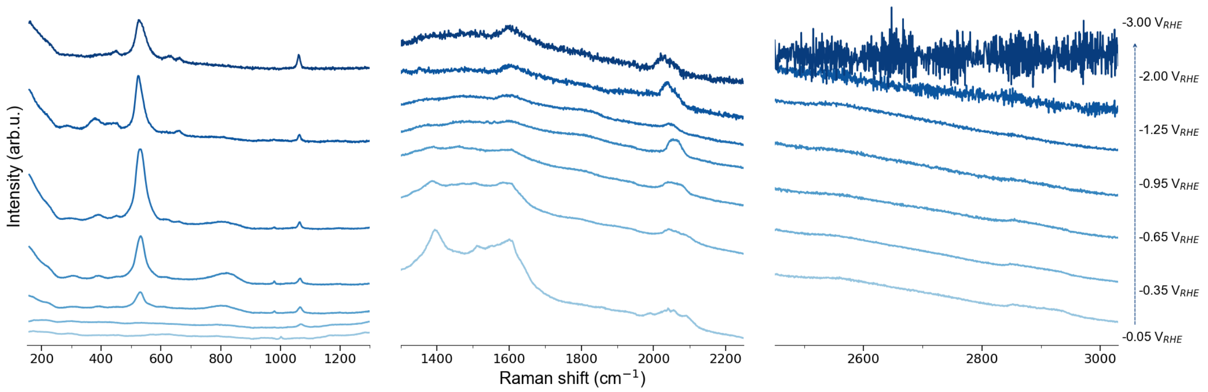

Figure S4: In situ Raman spectra of Cu/PFSA at different potentials in 0.5M K_2SO_4 + H_2SO_4 at pH 4.

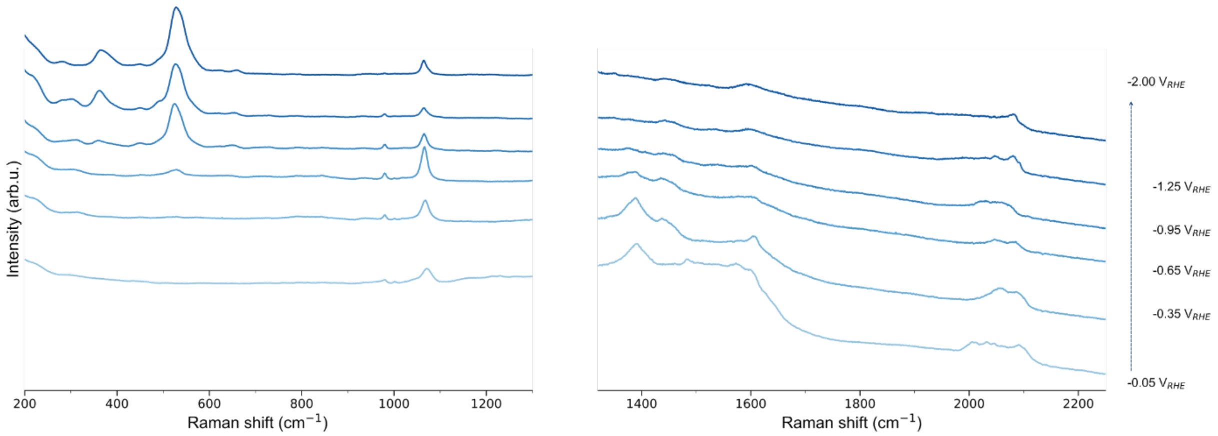

Figure S5: In situ Raman spectra of Cu/PFSA at different potentials in 0.5M K_2SO_4 without addition of H_2SO_4.

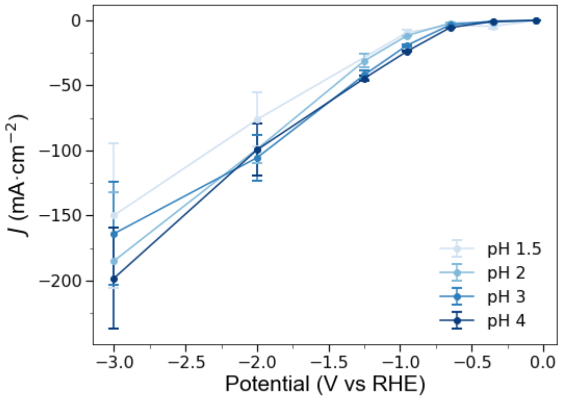

Figure S6: Current Density vs Potential of in situ SERS experiments at different pHs.

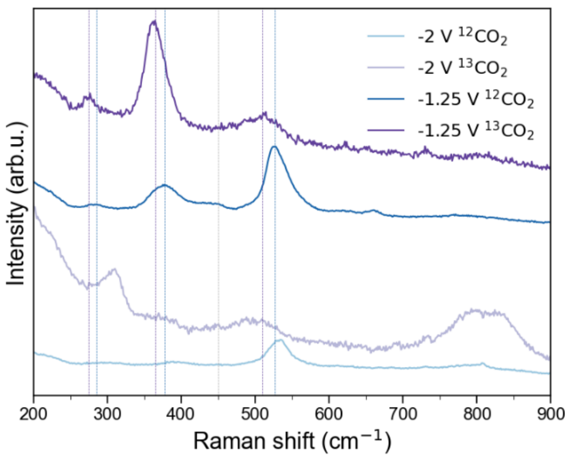

Figure S7: In situ Raman spectra of Cu/PFSA at different potentials in 0.5M K_2SO_4 + H_2SO_4 at pH 2 with $^{12}CO_2$ (blue) and $^{13}CO_2$ (purple).

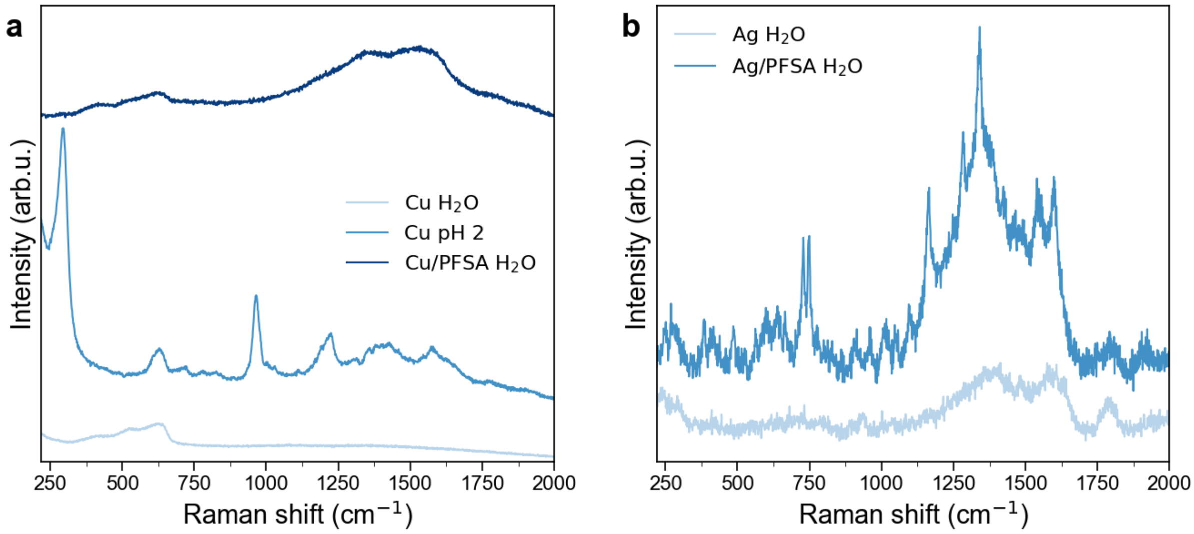

Figure S8: (a) Raman spectra of Cu in water (light blue), in K_2SO_4 + H_2SO_4 at pH 2 (blue), and Cu/PFSA in water (dark blue). (b) Raman spectra of Ag (light blue) and Ag/PFSA (blue) in water. Traces show a contribution from ionomer to 1300-1600 cm^{-1} peaks in absence of potential or CO_2 feed. Additionally other peaks from carbonate and other *O-C-(X) species overlap in this region (panel a, Cu pH 2 trace).[1]

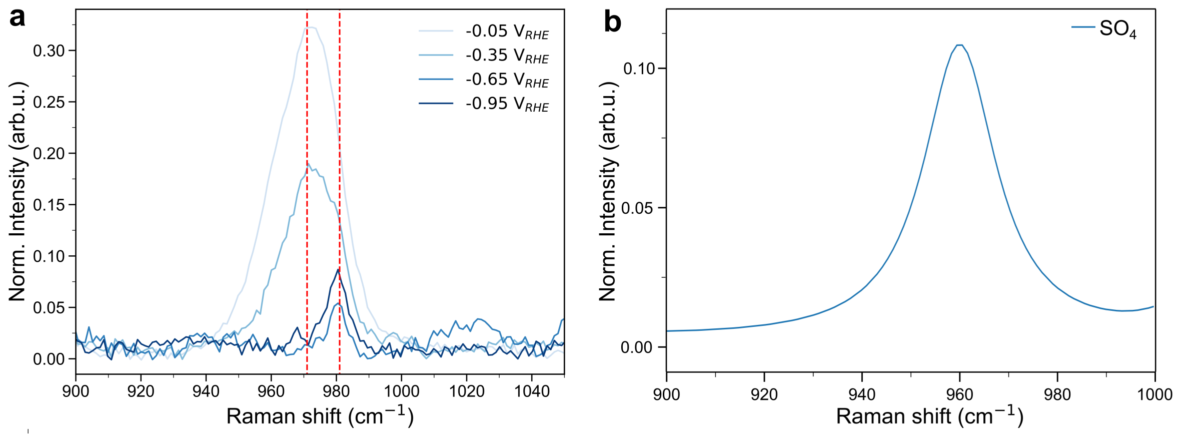

Figure S9: (a) Spectra traces examples of sulfate peaks at different potentials, showing the 2 distinct peaks around 970 and 980 cm^{-1}. (b) Theoretical Raman spectra of sulfate showing a peak around 960 cm^{-1} at 0 V_{RHE}.

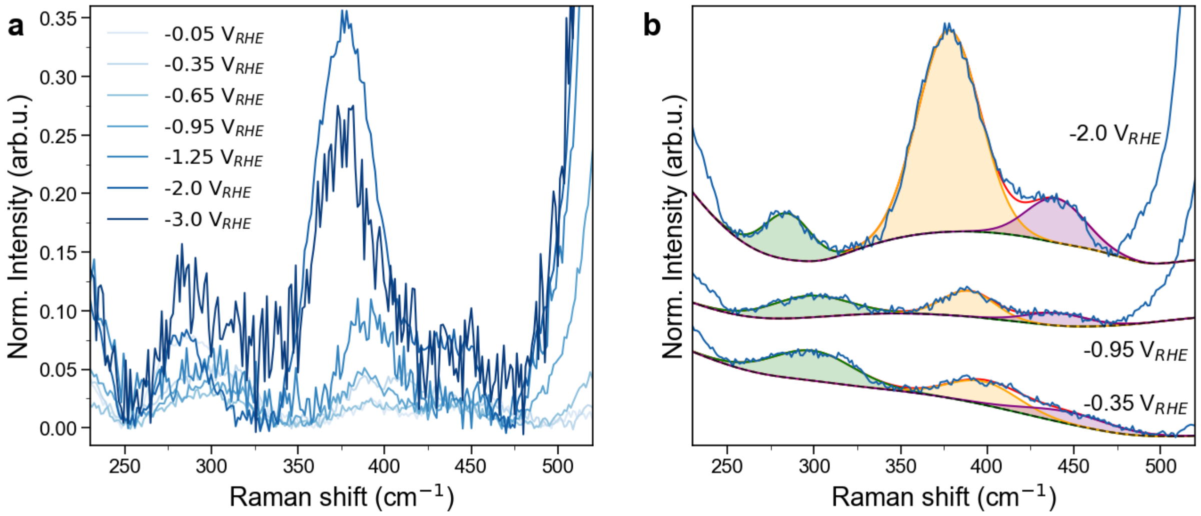

Figure S10: (a) Spectra traces examples for peaks in 250-480 cm^{-1} range at different potentials showing evolution of Cu-C(O) stretching and frustrated rotation CO bands ratio. (b) Fitting examples for spectra in 3 different potentials.

CO intermediate peaks

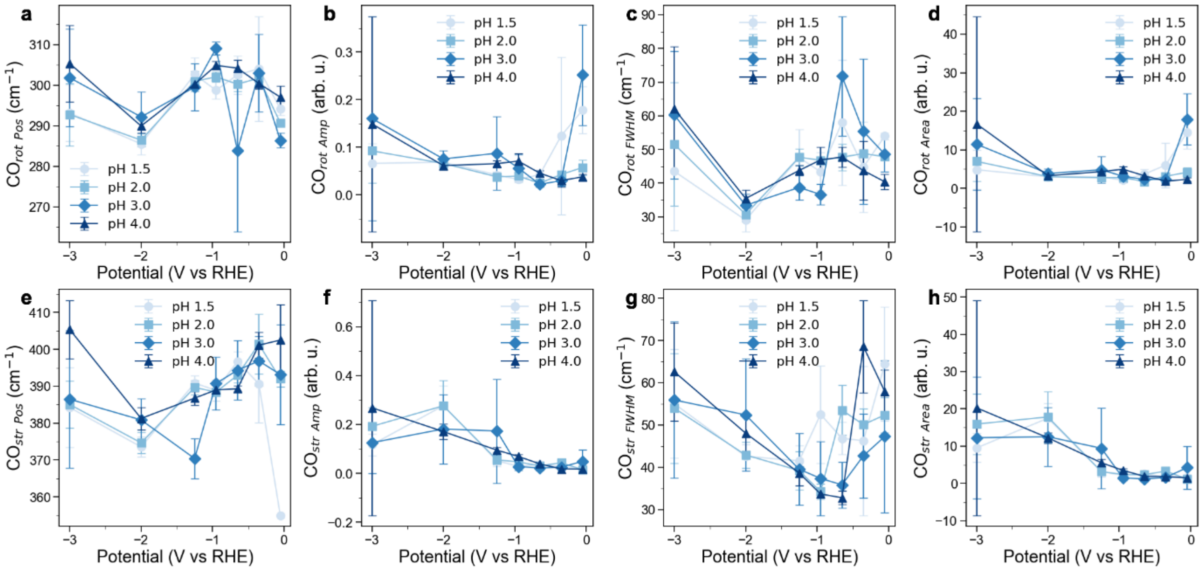

Figure S11: Position (a, e), amplitude (b, f), Full Width at Half Maximum (FWHM) (c, g) and area (d, h) of frustrated rotation CO peak (a-d) and Cu-CO stretching peak (e-h).

Hydroxyl peak

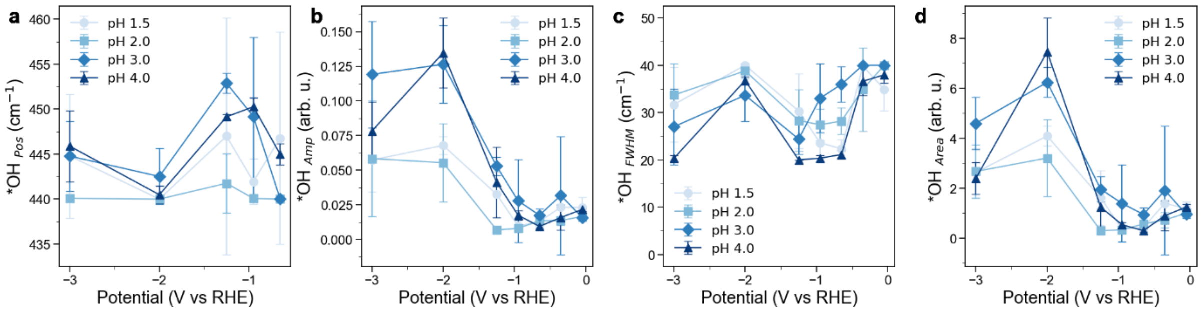

Figure S12: Position (a), amplitude (b), FWHM (c) and area (d) of hydroxyl peak.

*C-intermediate peak

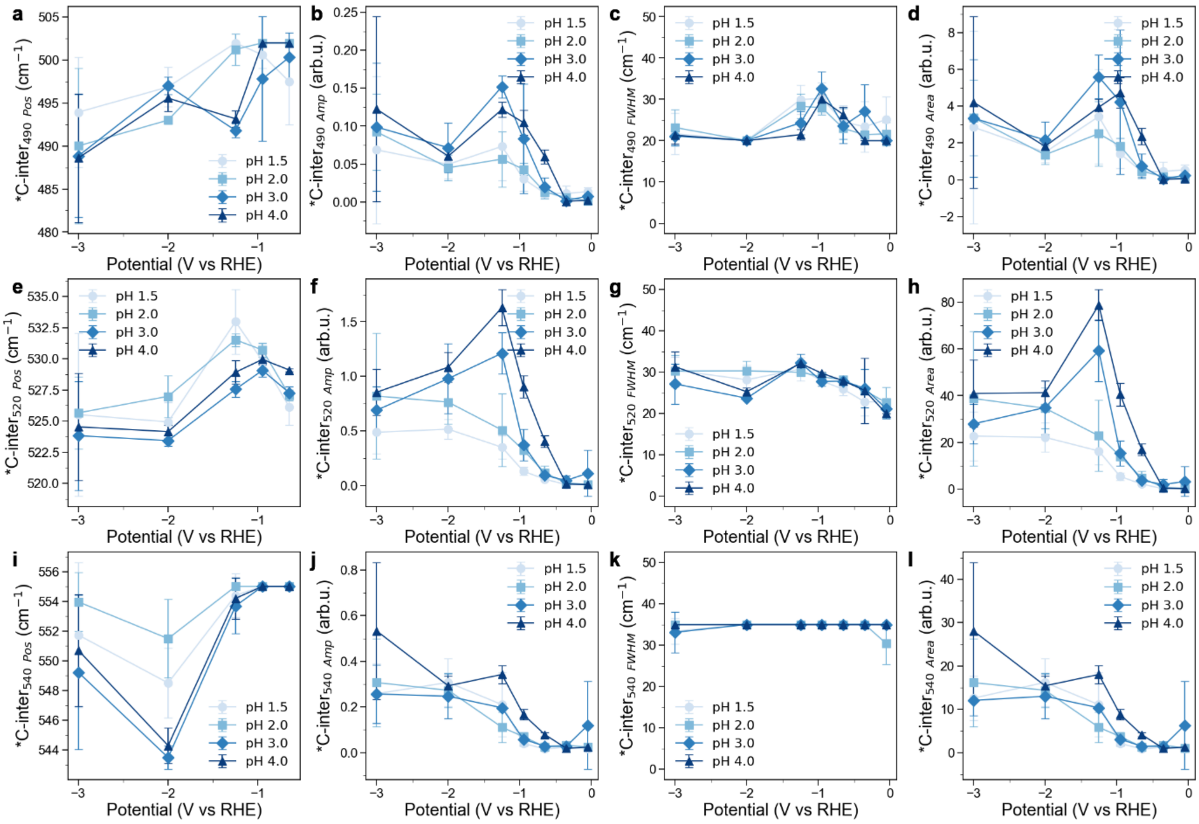

Figure S13: Position (a, e, i), amplitude (b, f, j), FWHM (c, g, k) and area (d, h, l) of the three fitted *C-inter peaks around 490 (a-d), 520 (e-h) and 540 (i-l) cm^{-1}.

Carbonate peak

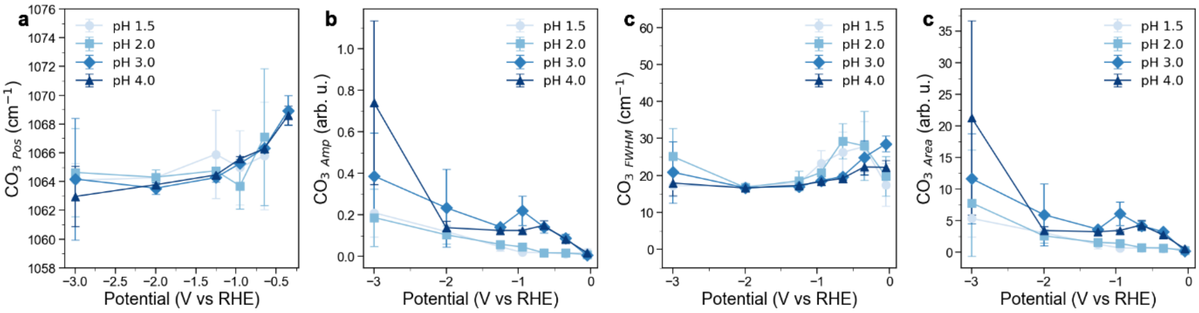

Figure S5: Position (a), amplitude (b), FWHM (c) and area (d) of carbonate peak fitting at different pHs.

Sulfate peak

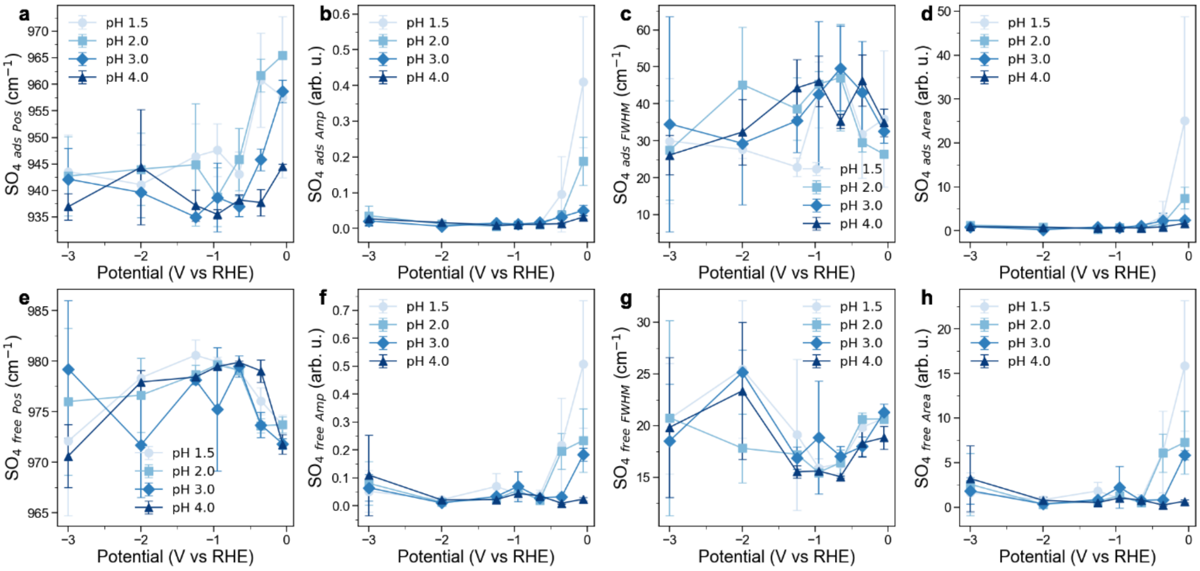

Figure S6: Position (a, e), amplitude (b, f), FWHM (c, g) and area (d, h) of adsorbed sulfate (a-d) and free sulfate (e-h) peaks.

Sulfate – Hydroxyl/oxide – CO peak correlation

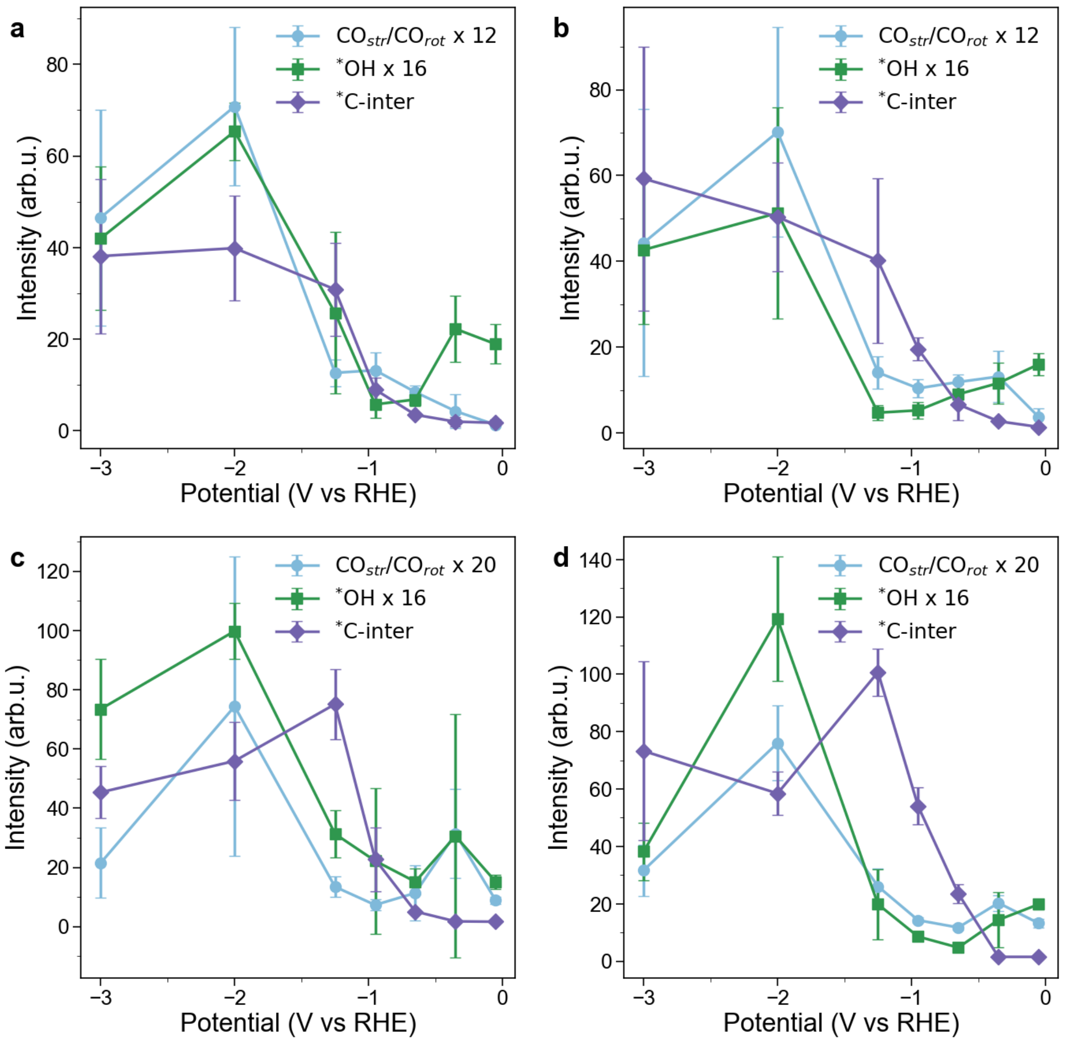

Figure S7: Comparative analysis of CO_2R-relevant species at pH 1.5 (a), 2 (b), 3 (c) and 4 (d), with scaled intensity of *C-intermediate (purple), str/rot CO band ratio (blue), OH_{bridge} (green) vs. potential.

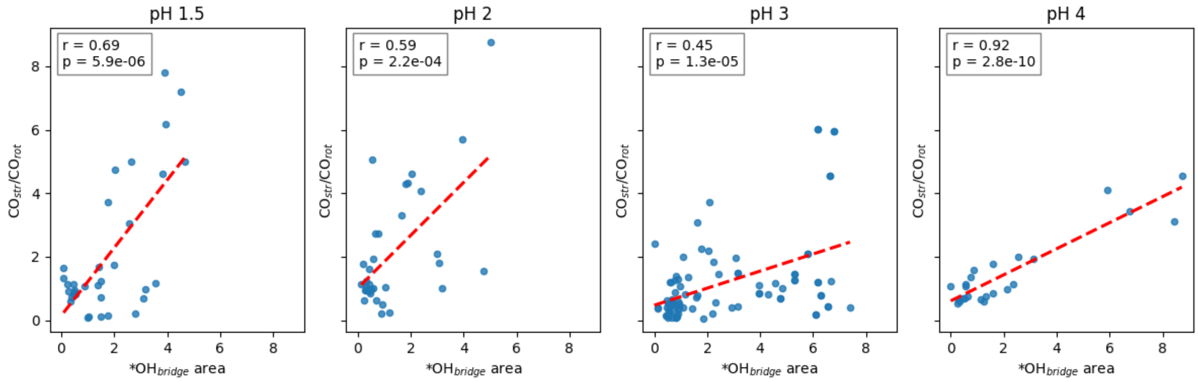

Figure S8: Scatter plot of str/rot CO ratio vs $*OH_{bridge}$ area for all potentials at different pHs (a to d).

Stochastic *CO bands (1900-2100 cm^{-1})

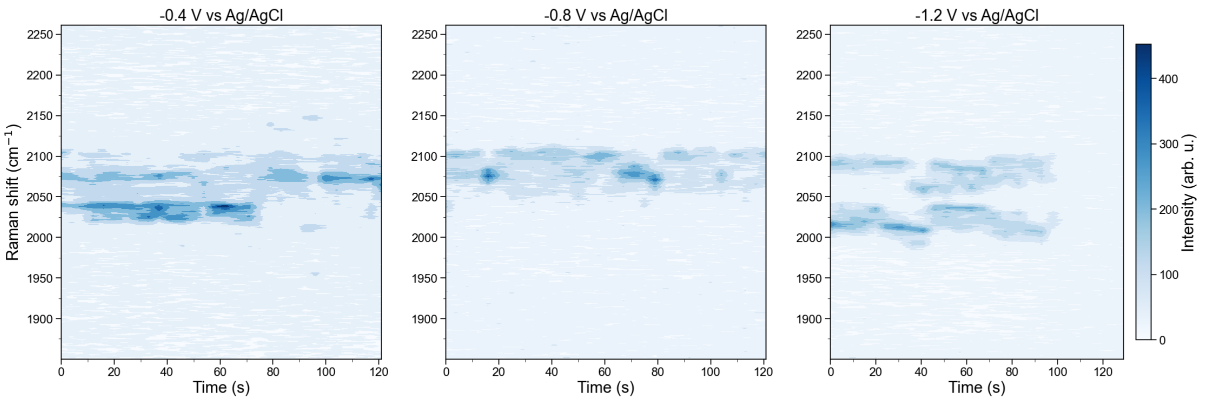

Figure S9: Time evolution traces examples of the *CO bands in the 1900-2100 cm^{-1} region for three different potentials (-0.4, -0.8 and -1.2 V vs Ag/AgCl).

Quasi-in situ XRD

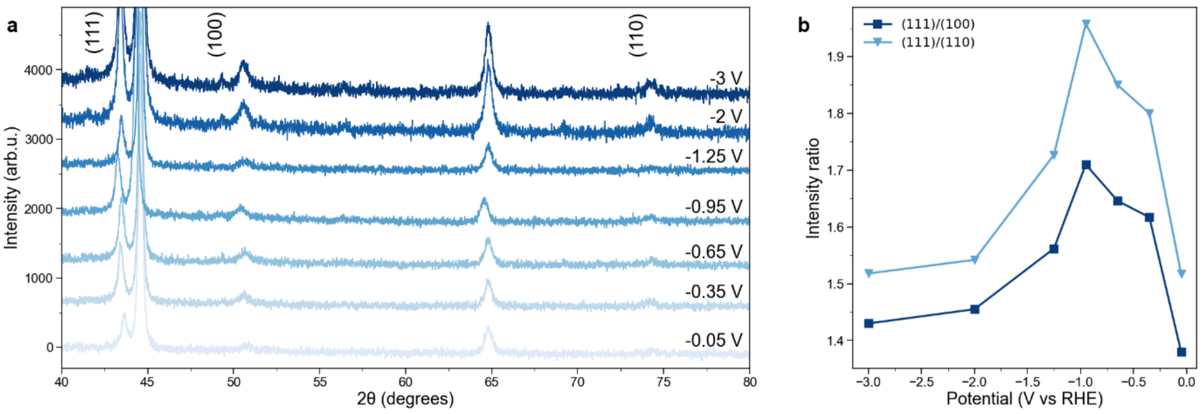

Figure S10: (a) Quasi-in situ X-ray diffraction patterns after applying a series of potentials, following the same procedure as for in situ Raman experiments, for Cu/PFSA at pH 2. (b) Intensity ratio between (111), and (100) [dark blue] or (110) [light blue].

Electrochemical performance

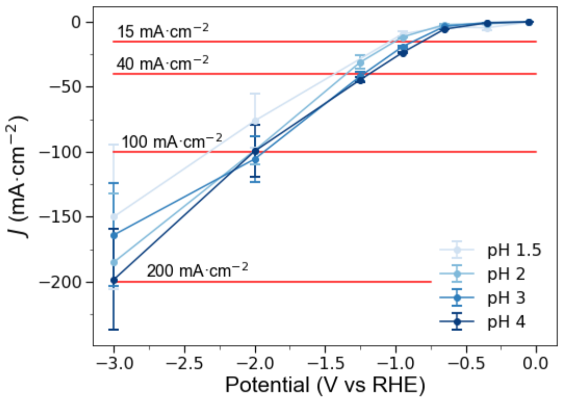

Figure S20: Current density vs potential of in situ SERS experiments at different pHs (blue lines). Red lines indicate the currents at which electrochemical test were performed.

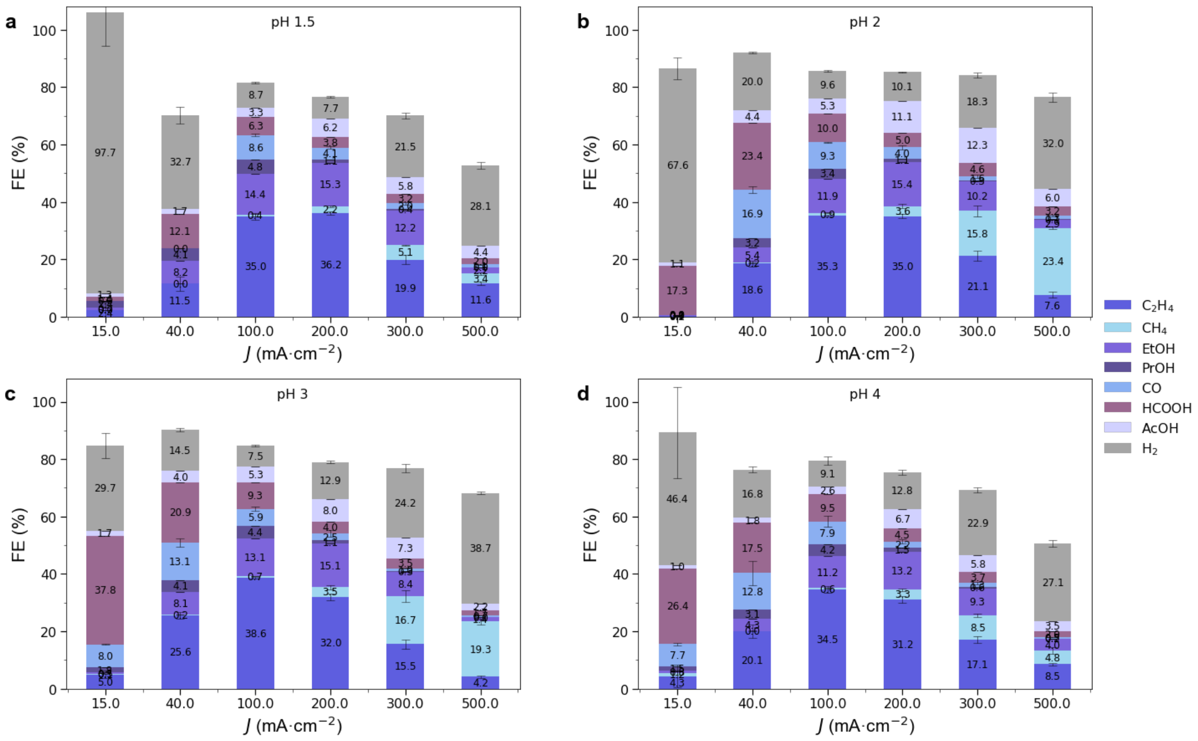

Figure S21: Faradaic efficiency towards H_2, and the different CO_2R products at different currents at pH 1.5 (a), pH 2 (b), pH 3 (c) and pH 4 (d). The remainder FE was verified to be H_2 produced at the catholyte chamber.

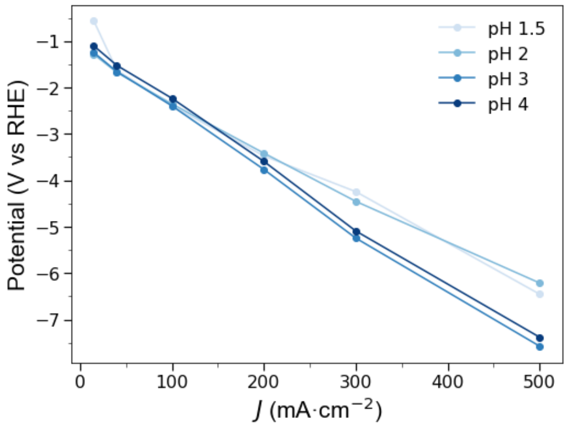

Figure S22: Potential vs current density of CO_2R electrochemical performance experiments at different pHs.

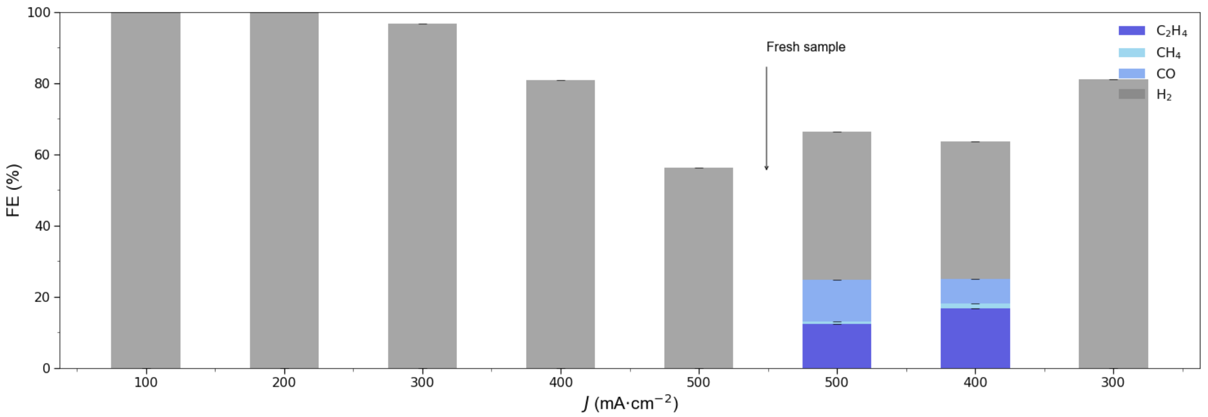

Figure S23: Faradaic efficiency towards H_2, C_2H_4, CH_4 and CO at different currents at pH 1. From left to right, experiments up to 500 mA·cm^{-2} were performed with a fresh sample. After that, fresh sample was used and currents were scanned starting from 500 to 300 mA·cm^{-2}. Experiment was carried out doing a single injection per point and without analysing liquid product.

The missing FE can be attributed mainly to H_2, and, to a lesser extent, to non-analysed liquid products. The deficit in H_2 FE is most likely due to H_2 crossover into the catholyte compartment. Consistent with this, gas analysis of the catholyte headspace revealed a dominant contribution from H_2.

Performance toward CO_2R products can be achieved at pH 1, although only at high current densities (>300 mA·cm^{-2}). Stability in such conditions is shown to be very poor (<30 min) given that product can only be observed at 400 mA·cm^{-2} when starting from high current densities, if starting from low current densities when reaching 400 mA·cm^{-2} sample shows already no catalytic activity towards CO_2R anymore.

DFT simulations

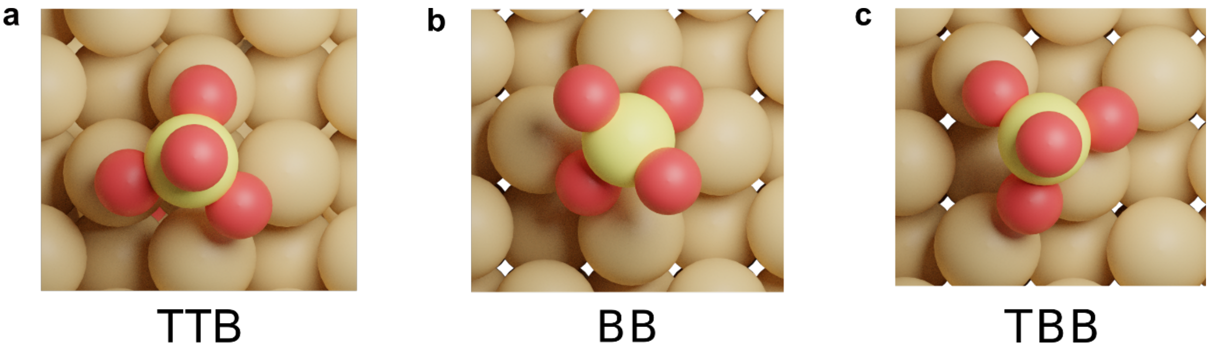

Figure S24: Adsorbate configurations of *SO_4 on Cu (100) a) TTB [Top, Top, Bridge] b) BB [Bridge, Bridge] c) TTB [Top, Top, Bridge].

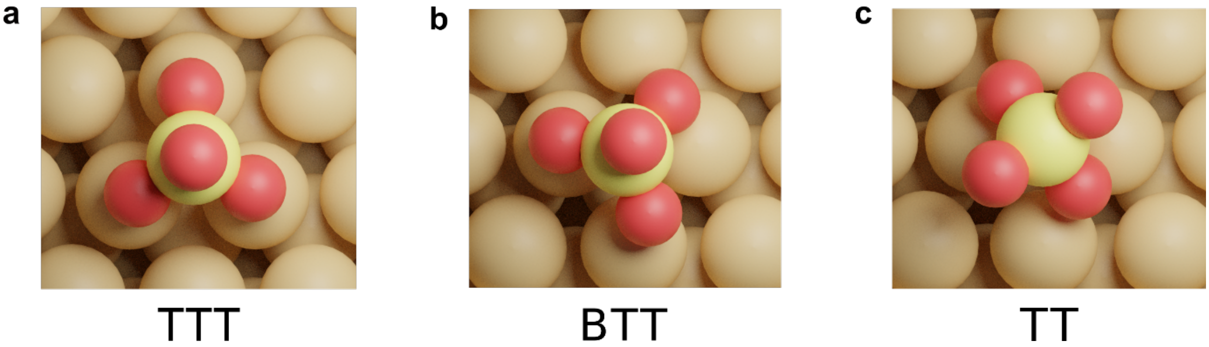

Figure S25: Adsorbate configuration of *SO_4 on Cu (111) a) TTT [Top, Top, Top] b) BTT [Bridge, Top, Top] c) TT [Top, Top].

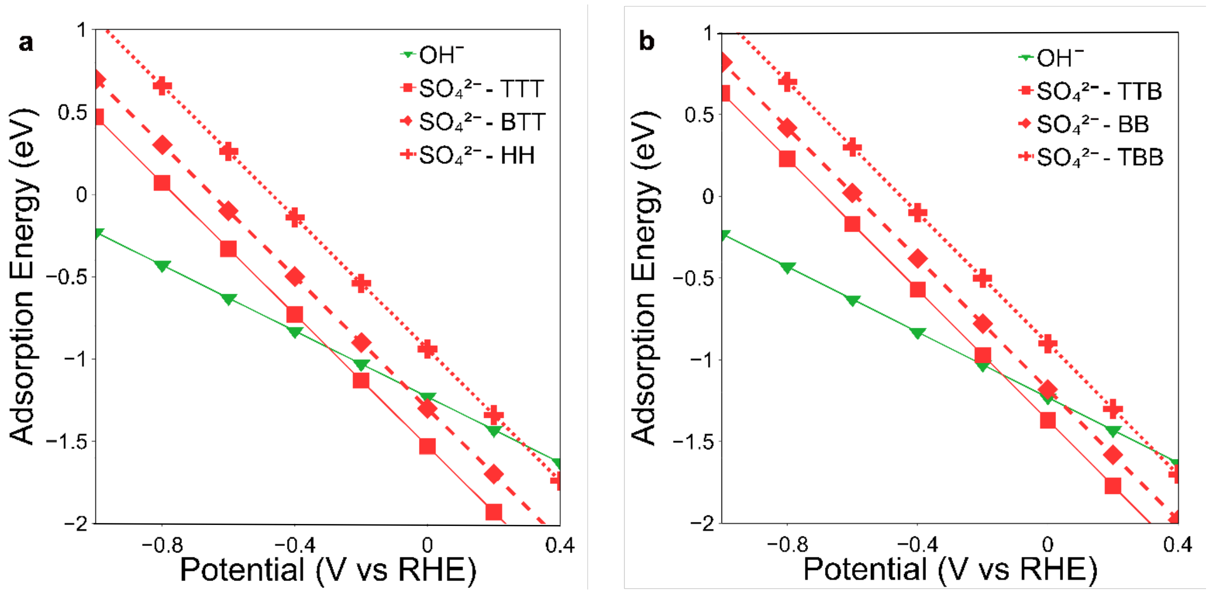

Figure S26: Adsorption energy of OH^- and different configurations of SO_4^{2-} computed using CHE approximation a) Cu (100) b) Cu (111) the configuration of all the are SO_4^{2-} mentioned in Figures S19 and S20.

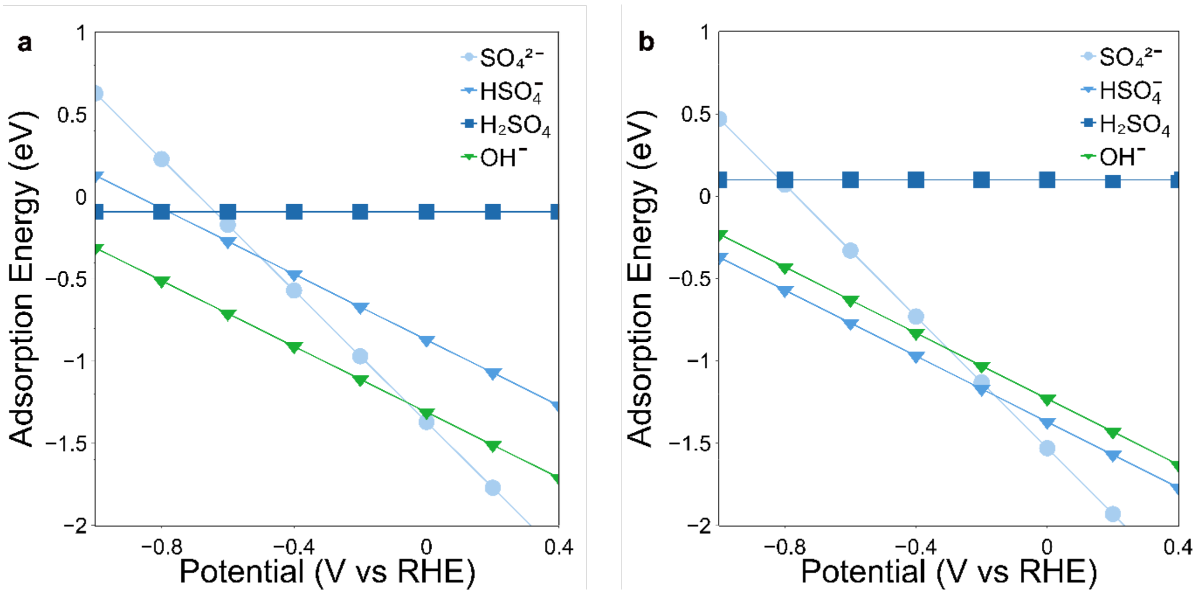

Figure S27: Adsorption energy of SO_4^{2-}, HSO_4^-, H_2SO_4, OH^- computed using CHE approximation a) Cu (100) b) Cu (111).

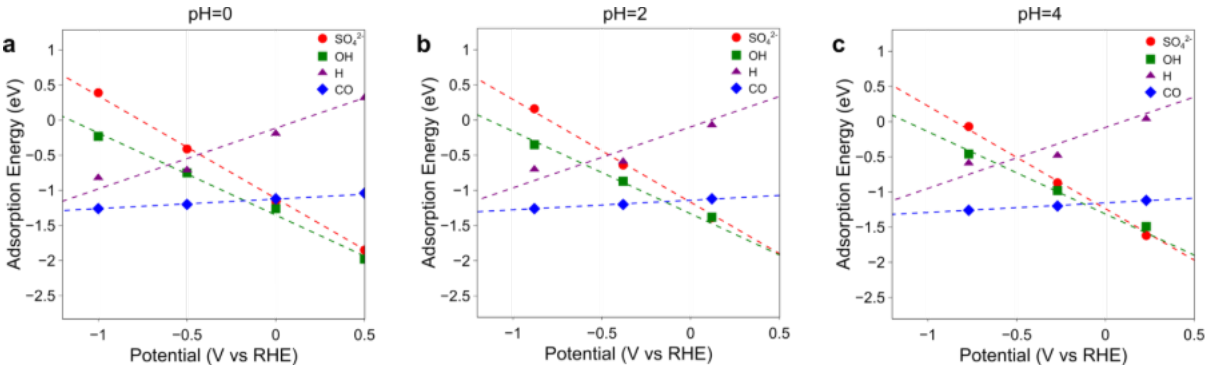

Figure S28: Adsorption energy of SO_4^{2-}, OH^-, H^+ and CO as function of potential calculated using GC-DFT on Cu(100).

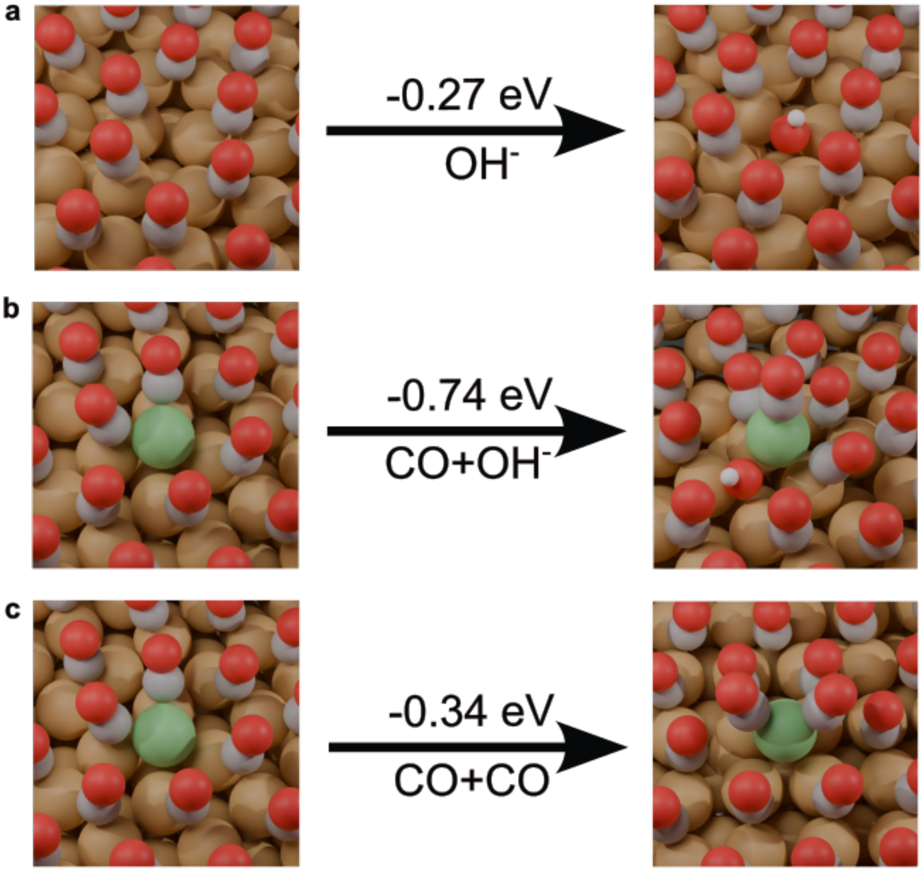

Figure S29: Adsorption energy of a) *OH on 0.85 ML of *CO on Cu(100) b) *CO and *OH on Cu adatom (marked as green) c)*CO-*CO geminal structures.

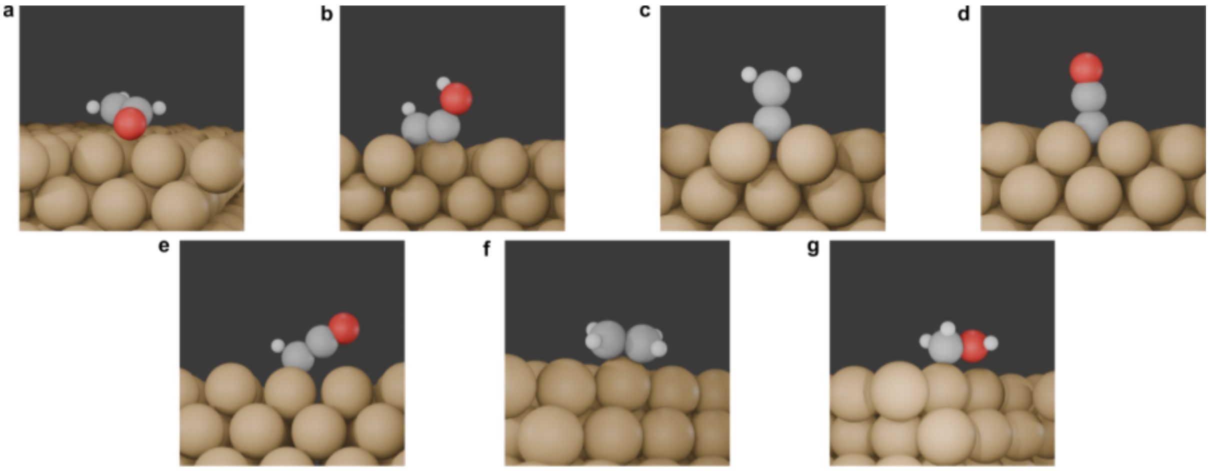

Figure S30: Adsorption configuration of C_2 and C_1 intermediates with frequency modes in the range of 510 and 550 cm^{-1}. The structures were taken from Pablo-García et al.[2] and rerun with our computational settings. a)*CH_2CHO b)*CHCOH c)*CCH_2 d)*CCO e)*CHCO f)*CH_2CH_2 g)*CH_2OH.

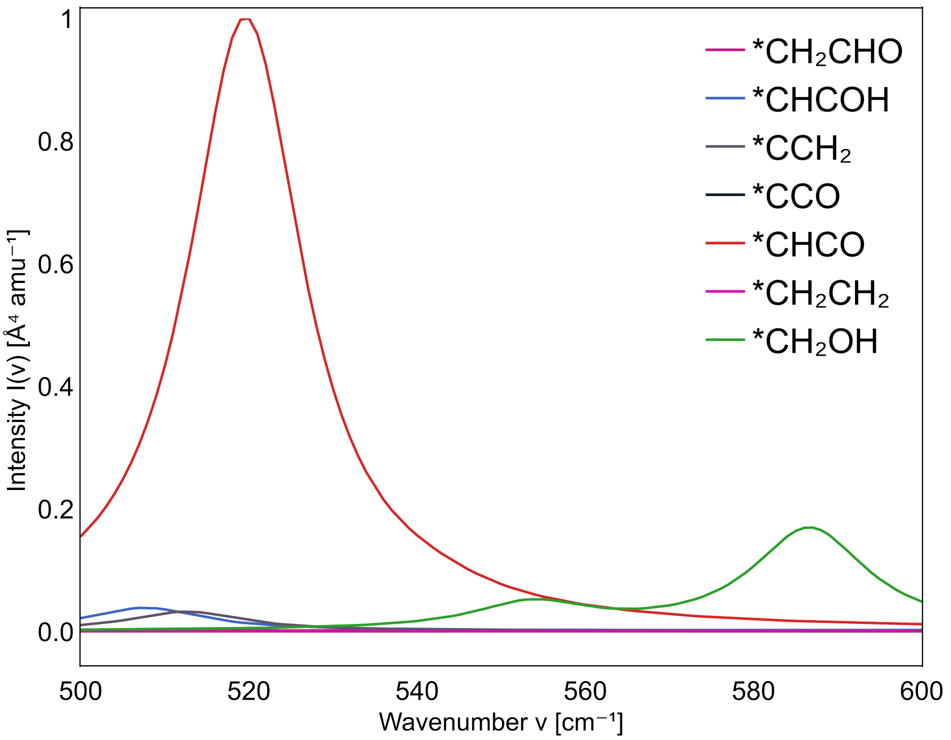

Figure S31: Theoretical Raman spectra of C_2 and C_1 intermediates with frequency modes in the range of 510 and 550 cm-1 taken from Pablo-García et al.[2]

Table S1: Computed frequencies associated with adsorbate binding configurations on Cu(100).

Species	Vibration	Direction of vibration	Frequency (cm^{-1})
*SO_4 -TTB	Symmetric stretching		978
*OH_{Hollow}	Cu-O-H bending	Along X direction	663
		Along Y direction	649
*OH_{Bridge}	Cu-O-H bending	Along X direction	527
		Along Y direction	674
CH_2CHO*	O-C-C bending	In XY plane	510

Table S2: Computed frequencies associated with adsorbate binding configurations on Cu(111).

Species	Vibration	Direction of vibration	Frequency (cm^{-1})
*SO_4 -TTT	Symmetric stretching		950
*OH_{Hollow}	Cu-O-H bending	Along X direction	561
		Along Y direction	547
*OH_{Bridge}	Cu-O-H bending	Along X direction	452
		Along Y direction	436
CH_2CHO*	O-C-C bending	In XY plane	500

Table S3: Computed frequencies associated with adsorbate binding configurations on Cu(100) with adatom.

Species	Vibration	Direction of vibration	Frequency (cm^{-1})
*CO	CO top stretching		2114
*OH	Cu-O stretching	Along X direction	433
		Along Y direction	324

Table S4: Adsorption energies of *SO_4 configurations on Cu(100), the configurations are defined in Figure S24.

Config	Adsorption energy (eV)
TTB	-1.37
BB	-1.18
TBB	-0.90

Table S5: Adsorption energies of *SO_4 configurations on Cu(111), the configurations are defined in Figure S25.

Config	Adsorption energy (eV)
TTT	-1.53
BTT	-1.30
HH	-0.94

Table S6: Frequency modes associated with C_1 and C_2 intermediates on Cu (100) in the range of 510–550 cm^{-1} taken from Pablo-García et al.[2]

Species	Frequency (cm^{-1})
*CH_2CHO	511
*CHCHO	529
*CCH_2	523
*CCO	534
*CHCO	528
*C_2H_4	518
*CH_2OH	554

Derivation for adsorption energy of anions

Sulfates:

Adsorption energy of SO_4^{2-} was calculated by following reaction:

$$2H^+ + SO_4^{2-} + * \longrightarrow SO_4 * + 2H^+ + 2e^- \quad (1)$$

Adsorption energy:

$$BE_{SO_4^{-2}} = E_{SO_4*} + 2(\frac{1}{2}E_{H_2}(g) - V_{RHE}) - E_{[2H^+ + SO_4^{2-}]} - E_* \quad (2)$$

E_{SO_4*}, E_* are calculated using DFT and $\frac{1}{2}E_{H_2}(g) - V_{RHE}$ is derived from computational hydrogen electrode approximation. To estimate the energy of $E_{[2H^+ + SO_4^{2-}]}$ using DFT, we consider the acidic dissociation of H_2SO_4.

$$H_2SO_4(aq) \longrightarrow 2H^+ + SO_4^{2-} \quad (3)$$

$$E_{[2H^+ + SO_4^{2-}]} = E_{H_2SO_4}(aq) + 2.303k_BT(pKa_1 + pKa_2) \quad (4)$$

To get the $E_{H_2SO_4}$(aq) energy, we consider the following equilibrium:

$$H_2SO_4(aq) \leftrightharpoons H_2SO_4(g) \quad (5)$$

$$E_{H_2SO_4}(aq) = E_{H_2SO_4}(g) + k_BT\ln\left(\frac{p_{H_2SO_4}}{p_o}\right) \quad (6)$$

Where $p_{H_2SO_4}$ is the partial pressure of H_2SO_4 and $E_{H_2SO_4}$(g) is the DFT gas phase energy.

Combining equation (6) with (4), the equation (2) can now be written a:

$$BE_{SO_4^{-2}} = E_{SO_4*} + E_{H_2}(g) - 2V_{RHE} - E_{H_2SO_4}(g) - 2.303k_BT(pKa_1 + pKa_2) - k_BT * \ln\left(\frac{p_{H_2SO_4}}{p_o}\right) - E_* - \delta_{Sol} \quad (7)$$

$$\delta_{Sol} = \delta_* - \delta_{SO_4*} \quad (8)$$

δ_{Sol} the solvation correction term calculated using implicit solvent method

Hydroxide

Adsorption energy of OH^- was calculated by the following reaction:

$$H^+ + OH^- + * \longrightarrow OH* + H^+ + e^- \quad (9)$$

Adsorption energy

$$BE_{OH^-} = E_{OH*} + \frac{1}{2}E_{H_2}(g) - V_{RHE} - E_{[H^+ + OH^-]} - E_*$$
(10)

E_{OH*} and E_* are calculated using DFT and $\frac{1}{2}E_{H_2}(g) - V_{RHE}$ is derived from computational hydrogen electrode approximation. To estimate the energy of $E_{[H^+ + OH^-]}$ using DFT, we will consider the water dissociation of H_2O

$$H_2O(aq) \longrightarrow H^+ + OH^- \quad (11)$$

$$E_{[H^+ + OH^-]} = E_{H_2O}(aq) + 2.303k_BTpKa \quad (12)$$

To get the E_{H_2O}(aq) energy, we consider the following equilibrium:

$$H_2O(aq) \leftrightharpoons H_2O(g) \quad (13)$$

$$E_{H_2O}(aq) = E_{H_2O}(g) + k_BT\ln\left(\frac{p_{H_2O}}{p_o}\right) \quad (14)$$

Combining equation (14) with (12),

$$E_{[H^+ + OH^-]} = E_{H_2O} + 2.303k_B T(pKa) + k_B T * \ln\left(\frac{p_{H_2O}}{p_o}\right) \quad (15)$$

Where p_{H_2O} is the partial pressure of water and E_{H_2O}(g) is the DFT gas phase energy.

Now using equation (15) in equation (10) can now be written as:

$$BE_{OH^-} = E_{OH*} + \frac{1}{2}E_{H_2} - V_{RHE} - E_{H_2O} - 2.303k_B T(pKa) - k_B T * \ln\left(\frac{p_{H_2O}}{p_o}\right) - E_* - \delta_{Sol} \quad (16)$$

$$\delta_{Sol} = \delta_* - \delta_{OH*} \quad (17)$$

δ_{Sol} is the solvation correction term calculated using implicit solvent method.

<u>Adsorption of OH on 0.85 ML CO on Cu(100)</u>

The adsorption energy of *OH in the presence of 0.85ML of CO on is as follows:

$$H^+ + OH^- + *_{nCO} \longrightarrow *_{nCO}\, OH + H^+ \quad (18)$$

$$BE_{*_{nCO}OH} = E_{*_{nCO}OH} + \frac{1}{2}E_{H_2}(g) - nE_{CO}(g) - E_{[H^+ + OH^-]} - E_{*_{nCO}} - \delta_{Sol} \quad (19)$$

Where $E_{*_{nCO}OH}$ is the energy of slab with *OH with n *CO adsorbed in the adatom, $E_{*_{ada}}$ is the energy of slab with Cu adatom, E_{H_2} is the energy of hydrogen, $E_{CO}(g)$is the energy of CO in gas phase, $E_{[H^+ + OH^-]}$ is the energy of $H^+ + OH^-$ which can be referred from equation (15) and δ_{Sol} is the solvation correction assumed to be same as the flat surface.

Absorption in the presence of adatom

<u>Co adsorption of CO and OH</u>

Adsorption energy of *CO and *OH in the presence of adatom is follows

$$H^+ + OH^- + CO + *_{ada} \longrightarrow CO *_{ada} OH + H^+ \quad (20)$$

The adsorption energy of *OH and *CO is calculated as follows,

$$BE_{*OH*CO} = E_{CO*_{ada}OH} + \frac{1}{2}E_{H_2}(g) - E_{CO}(g) - E_{[H^+ + OH^-]} - E_{*_{ada}} - \delta_{Sol} \quad (21)$$

Where $E_{CO*_{ada}OH}$ is the energy of slab with *OH and *CO adsorbed in the adatom, $E_{*_{ada}}$ is the energy of slab with Cu adatom, E_{H_2} is the energy of hydrogen, $E_{CO}(g)$is the energy of CO in gas phase, , $E_{[H^+ + OH^-]}$ is the energy of $H^+ + OH^-$ which can be referred from equation (15) and δ_{Sol} is the solvation correction assumed to be same as the flat surface.

<u>Adsorption of CO-CO</u>

Adsorption energy of 2 *CO in the presence of adatom is follows

$$2CO + *_{ada} \longrightarrow COCO *_{ada} OH + H^+ \quad (22)$$

The adsorption energy of 2 *CO is calculated as follows,

$$BE_{*OH*CO} = E_{COCO*_{ada}} - 2\,E_{CO}(g) - E_{*_{ada}} - \delta_{Sol} \tag{22}$$

Where $E_{COCO*_{ada}}$ is the energy of slab with 2 *CO adsorbed in the adatom, $E_{*_{ada}}$ is the energy of slab with Cu adatom, $E_{CO}(g)$is the energy of CO in gas phase and δ_{Sol} is the solvation correction assumed to be same as the flat surface.

Note S1: Sulfate peaks

At low overpotentials, the in situ SERS spectra display two well-resolved bands at approximately 980 and 970 cm^{-1} (**Figure S9**), which are associated with sulfate anions. This spectral splitting and 10-15 cm^{-1} redshift is consistent with sulfate existing in both surface-adsorbed and interfacial (outer-sphere) configurations.[3–5]

Furthermore, the pH dependent trends in both adsorbed and interfacial sulfate further strengthens this interpretation. This pH-dependent behavior is consistent with the established behavior of sulfate inner- and outer-sphere species on other metal and metal oxide surfaces, where surface-adsorbed sulfate coverage decreases with increasing pH due to competition with hydroxide ions.[6–8]

Note S2: 450 cm^{-1} peak

Although the intensity of the hydroxyl peak at 450 cm^{-1} is relatively low compared to the adjacent *CO and *C-intermediate peaks, it can be clearly differentiated as a separate surface vibration mode, especially visible at higher potentials (-2 to -3 V_{RHE}, **Figure S1-5**). The fitting parameters of this feature are reproducible across different spots of the catalyst surface (**Figure S8**), and remain consistent across experiments performed at different pH values.

While *OH contributions to the feature at 530 cm^{-1} cannot be ruled out,[9–15] as suggested by our DFT calculations (**Table S1**), the results from $^{13}CO_2$ labelling experiment indicate that the main contributor to this feature is carbon-containing species.[16–18]

References

(1) Zhan, C.; Dattila, F.; Rettenmaier, C.; Herzog, A.; Herran, M.; Wagner, T.; Scholten, F.; Bergmann, A.; López, N.; Roldan Cuenya, B. Key Intermediates and Cu Active Sites for CO2 Electroreduction to Ethylene and Ethanol. *Nat. Energy* **2024**, *9* (12), 1485–1496. https://doi.org/10.1038/s41560-024-01633-4.

(2) Pablo-García, S.; Veenstra, F. L. P.; Ting, L. R. L.; García-Muelas, R.; Dattila, F.; Martín, A. J.; Yeo, B. S.; Pérez-Ramírez, J.; López, N. Mechanistic Routes toward C_3 Products in Copper-Catalysed CO_2 Electroreduction. *Catal. Sci. Technol.* **2022**, *12* (2), 409–417. https://doi.org/10.1039/D1CY01423D.

(3) Niaura, G.; Malinauskas, A. Surface-Enhanced Raman Spectroscopy of ClO_4^- and SO_4^{2-} Anions Adsorbed at a Cu Electrode. *J. Chem. Soc. Faraday Trans.* **1998**, *94* (15), 2205–2211. https://doi.org/10.1039/A800574E.

(4) Smoliński, S.; Sobkowski, J. Adsorption of Sulfate Ions on Monocrystalline Copper Electrodes: The Structural Effects. *J. Electroanal. Chem.* **1999**, *463* (1), 1–8. https://doi.org/10.1016/S0022-0728(98)00424-0.

(5) Brown, G. M.; Hope, G. A. A SERS Study of $SO^{-2}{}_4Cl^-$ Ion Adsorption at a Copper Electrode in-Situ. *J. Electroanal. Chem.* **1996**, *405* (1), 211–216. https://doi.org/10.1016/0022-0728(95)04400-0.

(6) Wang, X.; Wang, Z.; Peak, D.; Tang, Y.; Feng, X.; Zhu, M. Quantification of Coexisting Inner- and Outer-Sphere Complexation of Sulfate on Hematite Surfaces. *ACS Earth Space Chem.* **2018**, *2* (4), 387–398. https://doi.org/10.1021/acsearthspacechem.7b00154.

(7) Gu, C.; Wang, Z.; Kubicki, J. D.; Wang, X.; Zhu, M. X-Ray Absorption Spectroscopic Quantification and Speciation Modeling of Sulfate Adsorption on Ferrihydrite Surfaces. *Environ. Sci. Technol.* **2016**, *50* (15), 8067–8076. https://doi.org/10.1021/acs.est.6b00753.

(8) Zhang, G. Y.; Peak, D. Studies of Cd(II)–Sulfate Interactions at the Goethite–Water Interface by ATR-FTIR Spectroscopy. *Geochim. Cosmochim. Acta* **2007**, *71* (9), 2158–2169. https://doi.org/10.1016/j.gca.2006.12.020.

(9) Wu, Y.; Chen, C.; Liu, S.; Qian, Q.; Zhu, Q.; Feng, R.; Jing, L.; Kang, X.; Sun, X.; Han, B. Highly Selective CO_2 Electroreduction to Multi-Carbon Alcohols via Amine Modified Copper Nanoparticles at Acidic Conditions. *Angew. Chem. Int. Ed.* **2024**, *63* (49), e202410659. https://doi.org/10.1002/anie.202410659.

(10) Lei, Q.; Huang, L.; Yin, J.; Davaasuren, B.; Yuan, Y.; Dong, X.; Wu, Z.-P.; Wang, X.; Yao, K. X.; Lu, X.; Han, Y. Structural Evolution and Strain Generation of Derived-Cu Catalysts during CO_2 Electroreduction. *Nat. Commun.* **2022**, *13* (1), 4857. https://doi.org/10.1038/s41467-022-32601-9.

(11) Ma, H.; Ibáñez-Alé, E.; You, F.; López, N.; Yeo, B. S. Electrochemical Formation of C_{2+} Products Steered by Bridge-Bonded *CO Confined by *OH Domains. *J. Am. Chem. Soc.* **2024**, *146* (44), 30183–30193. https://doi.org/10.1021/jacs.4c08755.

(12) Bodappa, N.; Su, M.; Zhao, Y.; Le, J.-B.; Yang, W.-M.; Radjenovic, P.; Dong, J.-C.; Cheng, J.; Tian, Z.-Q.; Li, J.-F. Early Stages of Electrochemical Oxidation of Cu(111) and Polycrystalline Cu Surfaces Revealed by in Situ Raman Spectroscopy. *J. Am. Chem. Soc.* **2019**, *141* (31), 12192–12196. https://doi.org/10.1021/jacs.9b04638.

(13) Chang, X.; Zhao, Y.; Xu, B. pH Dependence of Cu Surface Speciation in the Electrochemical CO Reduction Reaction. *ACS Catal.* **2020**, *10* (23), 13737–13747. https://doi.org/10.1021/acscatal.0c03108.

(14) Niaura, G. Surface-Enhanced Raman Spectroscopic Observation of Two Kinds of Adsorbed OH− Ions at Copper Electrode. *Electrochimica Acta* **2000**, *45* (21), 3507–3519. https://doi.org/10.1016/S0013-4686(00)00434-5.
(15) Shao, F.; Xia, Z.; You, F.; Wong, J. K.; Low, Q. H.; Xiao, H.; Yeo, B. S. Surface Water as an Initial Proton Source for the Electrochemical CO Reduction Reaction on Copper Surfaces. *Angew. Chem. Int. Ed.* **2023**, *62* (3), e202214210. https://doi.org/10.1002/anie.202214210.
(16) An, H.; de Ruiter, J.; Wu, L.; Yang, S.; Meirer, F.; van der Stam, W.; Weckhuysen, B. M. Spatiotemporal Mapping of Local Heterogeneities during Electrochemical Carbon Dioxide Reduction. *JACS Au* **2023**, *3* (7), 1890–1901. https://doi.org/10.1021/jacsau.3c00129.
(17) Shan, W.; Liu, R.; Zhao, H.; He, Z.; Lai, Y.; Li, S.; He, G.; Liu, J. In Situ Surface-Enhanced Raman Spectroscopic Evidence on the Origin of Selectivity in CO_2 Electrocatalytic Reduction. *ACS Nano* **2020**, *14* (9), 11363–11372. https://doi.org/10.1021/acsnano.0c03534.
(18) Yang, F.; Jiang, S.; Liu, S.; Beyer, P.; Mebs, S.; Haumann, M.; Roth, C.; Dau, H. Dynamics of Bulk and Surface Oxide Evolution in Copper Foams for Electrochemical CO_2 Reduction. *Commun. Chem.* **2024**, *7* (1), 66. https://doi.org/10.1038/s42004-024-01151-0.
